# Early Stopping in Experimentation With Real-Time Functional Magnetic Resonance Imaging Using a Modified Sequential Probability Ratio Test

**DOI:** 10.3389/fnins.2021.643740

**Published:** 2021-11-04

**Authors:** Sarah J. A. Carr, Weicong Chen, Jeremy Fondran, Harry Friel, Javier Sanchez-Gonzalez, Jing Zhang, Curtis Tatsuoka

**Affiliations:** ^1^Department of Neuroimaging, Institute of Psychiatry, Psychology and Neuroscience, King’s College London, London, United Kingdom; ^2^Department of Neurology, Case Western Reserve University, Cleveland, OH, United States; ^3^Department of Computer and Data Sciences, Case Western Reserve University, Cleveland, OH, United States; ^4^Department of Population and Quantitative Health Sciences, Case Western Reserve University, Cleveland, OH, United States; ^5^Philips Healthcare, Highland Heights, OH, United States; ^6^Philips Healthcare, Madrid, Spain

**Keywords:** real-time fMRI, adaptive fMRI, dynamic experimentation, SPRT (sequential probability ratio test), early stopping fMRI

## Abstract

**Introduction:** Functional magnetic resonance imaging (fMRI) often involves long scanning durations to ensure the associated brain activity can be detected. However, excessive experimentation can lead to many undesirable effects, such as from learning and/or fatigue effects, discomfort for the subject, excessive motion artifacts and loss of sustained attention on task. Overly long experimentation can thus have a detrimental effect on signal quality and accurate voxel activation detection. Here, we propose dynamic experimentation with real-time fMRI using a novel statistically driven approach that invokes early stopping when sufficient statistical evidence for assessing the task-related activation is observed.

**Methods:** Voxel-level sequential probability ratio test (SPRT) statistics based on general linear models (GLMs) were implemented on fMRI scans of a mathematical 1-back task from 12 healthy teenage subjects and 11 teenage subjects born extremely preterm (EPT). This approach is based on likelihood ratios and allows for systematic early stopping based on target statistical error thresholds. We adopt a two-stage estimation approach that allows for accurate estimates of GLM parameters before stopping is considered. Early stopping performance is reported for different first stage lengths, and activation results are compared with full durations. Finally, group comparisons are conducted with both early stopped and full duration scan data. Numerical parallelization was employed to facilitate completion of computations involving a new scan within every repetition time (TR).

**Results:** Use of SPRT demonstrates the feasibility and efficiency gains of automated early stopping, with comparable activation detection as with full protocols. Dynamic stopping of stimulus administration was achieved in around half of subjects, with typical time savings of up to 33% (4 min on a 12 min scan). A group analysis produced similar patterns of activity for control subjects between early stopping and full duration scans. The EPT group, individually, demonstrated more variability in location and extent of the activations compared to the normal term control group. This was apparent in the EPT group results, reflected by fewer and smaller clusters.

**Conclusion:** A systematic statistical approach for early stopping with real-time fMRI experimentation has been implemented. This dynamic approach has promise for reducing subject burden and fatigue effects.

## Introduction

Analysis of task-based fMRI scans is typically performed with fixed, predetermined experimental designs. As a result, subjects must often endure stimulus protocols that are overly long in order to ensure the neural activity can be statistically discerned in the noisy data. However, this can lead to fatigue, learning effects and excessive motion, such as from agitation, as well as being costlier to administer due to longer scan times and potentially less reliable measurement. Also, the experimenter does not know if the neural activity is detectable until long after the scanning session is over. Real-time functional MRI (RT-fMRI) provides an opportunity to ameliorate these issues. RT-fMRI has been successfully applied in the field of neurofeedback and biofeedback from neural responses, where subjects may be trained to alter their brain activity based on real-time information provided from the fMRI scans. This has been reported in ADHD (Alegria et al., 2017), healthy subjects with no psychiatric or neurological disorders (Lawrence et al., 2014; Sherwood et al., 2016), Alzheimer’s disease (Hohenfeld et al., 2017) and Parkinson’s disease (Subramanian et al., 2011, 2016). Its uses have also been described in psychoradiology to aid diagnosis and treatment planning in psychiatric disorders (Lui et al., 2016). Real-time resting state fMRI has for instance been studied and implemented as well using TurboFIRE (Vakamudi et al., 2020). A largely unexplored application of RT-fMRI is to dynamically and statistically determine when a stimulus has been sufficiently presented in terms of replication of blocks to terminate early. The magnitude of effort and variability in neural activity while completing a task will vary from person to person. Trial administration within a block design can be stopped early if sequentially updated statistical inference on activation can be determined with sufficient accuracy based on the observed BOLD (blood oxygen level dependent) signal response up to that point. This application will be explored in detail.

The benefits of adaptive RT-fMRI include: (1) Shorter scan times for fMRI testing: Shorter scan times cannot only save in technology and personnel costs, but fatigue and learning effects can be avoided, improving signal quality. Scanning becomes less burdensome on the subject as well, which is an especially important consideration for children or elderly subjects. (2) Real-time quality control: greater consistency in activation classification error can be obtained, through statistical error-based benchmarks for stopping rules and real-time feedback on classification performance and adjustment of stimulus durations. (3) Richer information: Paradigms can become more complex and sophisticated. With greater time efficiency and flexibility, more variations of a stimulus, such as reflected by a broader range of difficulty levels, can be administered in the same amount of time. (4) Wide applicability: Dynamic adjustment of stimuli based on BOLD response in real-time can be generally applied across a range of focus areas that investigate localization of brain activity, including cognition and motor functioning.

Since the advent of RT-fMRI in the mid 1990s (Cox et al., 1995), a handful of mainstream software packages have been developed for use by the fMRI community. These include Turbo BrainVoyager (Goebel, 2012), AFNI’s real-time plugin (Cox et al., 1995), FSL-based FRIEND (Sato et al., 2013) and Python-based OpenNFT (Koush et al., 2017). There have been a few previous studies that have employed adaptive task-based RT-fMRI. It has been used to determine ‘good’ and ‘bad’ brain states to optimize learning (Yoo et al., 2012); to determine a person’s brain state to judge their attention to a task (Debettencourt et al., 2015); to elicit activity in particular brain regions by presenting stimuli chosen based on the response to the previous stimulus (Lorenz et al., 2016); and to estimate when brain activity was mapped to a particular network (Lorenz et al., 2018). Each study used different methods to perform the real-time analysis. These include real-time general linear model (GLM) methods (Yoo et al., 2012; Lorenz et al., 2016), multivariate pattern analysis (Debettencourt et al., 2015) and a Bayesian optimization algorithm (Lorenz et al., 2018).

Here, we extend the use of a statistically based dynamic approach to RT-fMRI experimentation described in Feng et al. (2015), addressing issues related to practical implementation. This approach involves the sequential updating of voxel-level likelihood ratio tests, known as sequential probability ratio tests (SPRTs) and assessing after each scan whether there is sufficient statistical evidence to determine whether or not an associated parameter value indicates task activation. Such results, considered in aggregate across a collection of voxels, can be used as a basis for early stopping of experimentation. Most off-line, *post-hoc* analyses of fMRI data use the general linear model to test statistical associations of voxel activation magnitude to task administration (Cox, 1996; Lazar, 2008; Jenkinson et al., 2012). This approach involves the voxel-level estimation of task-related regression parameters that indicate magnitude of association between an expected hemodynamic response signal from a task and the observed BOLD signal. We have adapted this general method for real-time fMRI by incrementally updating GLM regression parameter estimates as soon as the brain volumes are collected. At the individual voxel level, we can then assess hypothesis tests related to activation that are based on these estimates. In aggregate, the voxel level analyses inform decisions on early stopping and the tailoring of fMRI experimentation (Feng et al., 2015).

In comparison to Feng et al. (2015), we adopt a two-stage estimation approach that allows for parameter values that represent activation thresholds to be formulated in terms of *z*-score scale at the voxel level. This adaptive specification avoids the intractable problem of pre-specifying magnitudes of GLM parameter values that would be considered as “active.” Such magnitudes need to be scaled relative to estimation variance, which should be stably estimated after a first stage. We determine an appropriate duration of the first stage by monitoring estimation convergence of key GLM parameters. Also, while in Feng et al. (2015) serial independence was assumed, here we use the “sandwich” estimator to recognize potential serial covariance in inference (Carroll et al., 1998; Kauermann and Carroll, 2001). The impact of early stopping on group analysis is considered here as well. Importantly, we now present a novel workflow to apply and implement these methods on a Philips scanner, with a dynamic feedback system that allows for real-time dynamic adjustment of the experimentation with subjects. This was facilitated with adoption of numerical parallelization techniques. This work supports the premise that adaptive, individualized experimentation is feasible and can lead to practical and useful savings in scan times by reducing experimental redundancy.

Another novel aspect of this work is the application of adaptive RT-fMRI in a sample group of 12 healthy adolescent subjects and 11 adolescents born extremely preterm (EPT). The fMRI stimulus was a mathematical version of the well-known 1-back task. Early stopping was implemented using sequential probability ratio test (SPRT) statistics and our server was a Linux workstation located in a nearby building. Processing of RT-fMRI was completed within 3 s, always before the next scan arrived. We observed time savings of up to 33% based on early stopping when 80% of voxels were classified, which equals up to 4-min savings with a 12-min scan. The impact on activation analysis from the selection of early stopping criteria is assessed, as described in detail below. Finally, as an illustration, we conduct a comparison of group analyses between difficulty levels for EPT and healthy control subjects, to assess the effects of early stopping in this context.

## Background Information

### General Linear Model

Briefly, the general linear model involves convolving a double gamma hemodynamic response function (HRF) with task indicator variables that denote timing of administration to reflect expected task-related BOLD responses. Voxel-level task-related regression parameters are estimated and represent the association of the observed response to expected task-activated BOLD signal. Thus, activation is assessed through statistical inference on regression parameters. For a given voxel up to time *t* (i.e., for scans 1 through *t*), the GLM takes the form:


(1.1) Y=tXβt+Et

where *Y*_*t*_ is a *t* × 1 vector of observed BOLD signal intensities for the voxel up to time *t*, *E*_*t*_ is a *t* × 1 vector that represents the error terms, *X*_*t*_ is a *t* × *p* design matrix and includes the expected BOLD signal values per condition and *β* = [*b*_1_…*b*_*j*_…*b*_*p*_]′ is a *p* × 1 regression coefficients vector. In this formulation, a regression parameter *b*_*j*_ can represent magnitude of association with condition j. *E*_*t*_ is assumed to be distributed as multivariate normal with mean zero and covariance *W*_*t*_, where *W*_*t*_ is a *t* × *t* covariance matrix. Error variances comprise the diagonal elements. For spatial correlation, we conduct spatial smoothing, so do not explicitly model the spatial correlation structure. *Y*_*t*_ is assumed to have a multivariate normal probability distribution as follows:


(1.2) $$\begin{array}{c} \;f\left(Y_{t},\beta ,W_{t}\right)\\ \;=\frac{1}{{\left(2\pi \right)}^{t/2}\left|W_{t}\right|}\text{exp}\left(-\frac{1}{2}{\left(Y_{t}-X_{t}\beta \right)}^{\prime }W_{t}^{-1}\left(Y_{t}-X_{t}\beta \right)\right) \end{array}$$

where | *W*_*t*_| is the determinant of *W*_*t*_. At each voxel, the focus is on estimation of *cβ*, where *c* is some 1 × *p* linear contrast. Hence, we consider the more general scenario when inferential interest can also be a linear combination of the task parameters, which includes the single task parameter case. The standard linear estimate for c*β* at time *t* is:


(1.3) cβt^=c(XtXt′)-1XtYt′

This estimator is normally distributed and unbiased under (1.2). We fit regression models in parallel for all voxels under consideration in a target region of interest (ROI), which could be a particular structure or include the whole brain. Real-time analysis requires signal and image processing steps, as well as the continual updating of statistical estimates as new scan data are received from the scanner. Hence, given the large number of voxels to be analyzed, real-time fMRI presents “big data” computational challenges.

### Sandwich Estimator

In our previous work (Feng et al., 2015), we assumed serial independence for computational simplicity. Here we recognize potential for more general correlation structure using the nonparametric sandwich estimator $\hat{var}\left[c\hat{\beta_{t}}\right]$ (Carroll et al., 1998; Kauermann and Carroll, 2001). The sandwich estimator is a robust, model-free variance estimator for $c\hat{\beta_{t}}$ that does not require assumptions such as homoscedastic error variance or serial independence. Importantly, it still provides asymptotically consistent variance estimates, although convergence rates can be slow (Carroll et al., 1998; Kauermann and Carroll, 2001). The approach is computationally feasible for real-time analysis. This is in contrast to our initial autoregressive modeling approaches for the covariance matrix *W*, which were too slow to estimate for real-time implementations given our available computational resources.

### Sequential Probability Ratio Test

At the voxel level, we can use the sequential analytic framework of Wald (1947); Wald and Wolfowitz (1948); Cox (1963); Tantaratana and Thomas (1977); Li (2010); Feng et al. (2015), to adaptively assess activation status using real-time fMRI. As we will demonstrate, Wald’s SPRT test statistic can serve as the basis of an efficient, incremental GLM testing approach that can greatly reduce the need for experimental block administrations compared with fixed designs while attaining similar classification performance in simulation, and activation patterns with subject data. This approach relies on a SPRT statistic to conduct hypothesis testing, with the null hypothesis representing no activation with respect to a task, and the alternative hypothesis representing some threshold of activation, as represented by a GLM parameter value (Feng et al., 2015). This statistic is updated with each new observation, and its value is compared with thresholds for stopping.

The general procedure of Wald’s SPRT is as follows. Consider a one-sided hypothesis of the form *H*_0_: *cβ* = *cβ*_0_ versus *H*_*a*_: *cβ* ≥ *cβ*_1_, where *c*(*β*_1_ − *β*_0_) > 0. Two-sided formulations are described in Wald (1947) and Feng et al. (2015). Implementation of Wald’s SPRT involves updating Wald’s likelihood ratio statistic as new data are observed (Wald, 1947):


(1.4) $$\Lambda_{t}=log\left(\frac{f\left(c\hat{\beta_{t}}|c\beta_{1},\hat{var}\left[c\hat{\beta_{t}}\right]\right)}{f\left(c{\hat{\beta_{t}}}_{t}|c\beta_{0},\hat{var}\left[c\hat{\beta_{t}}\right]\right)}\right)$$

where $f(c\hat{\beta_{t}}|c\beta_{0},\hat{var}\left[c\hat{\beta_{t}}\right]$) and $f\left(c\hat{\beta_{t}}|c\beta_{1},\hat{var}\left[c\hat{\beta_{t}}\right]\right)$are the respective normal probability densities functions of $c\hat{\beta_{t}}$ given *cβ*_0_ or *cβ*_1_ is the true value of the parameter of interest and conditioning on the estimated variance. After *Y*_*t*_ is observed up to time point, *t*, one of three possible decisions is made according to the following rules:

1. Continue sampling, if *B* < Λ_*t*_ < *A*
2. Stop sampling and accept *H*_0_, if Λ_*t*_ < *B*
3. Stop sampling and accept *H_a_*, if *A* < Λ_*t*_

where stopping boundaries are:


(1.5) $$\left(A,B\right)=\left(log\left(\frac{1-\beta_{E}}{\alpha_{E}}\right),log\left(\frac{\beta_{E}}{1-\alpha_{E}}\right)\right)$$

and the target Type I and Type II error levels are, respectively, denoted as α_E_ and *β*_E_. Note that both the Type I and Type II error levels are controlled for with SPRT, as opposed to standard hypothesis test formulations that only control for Type I error level. Multiple SPRTs are conducted concurrently across voxels. This can in principle be adjusted for by Bonferroni correction to account for this simultaneous testing. If the specified Type I error levels are too small, however, this can affect the feasibility of early stopping. Although not done here, it is possible to also consider *post-hoc* multiple comparisons methods after experimentation is completed (Lindquist and Mejia, 2015).

A practical modification of the original SPRT formulation for stopping is to consider the truncated SPRT (Sawasd Tantaratana, 1977), which will additionally call for stopping if an upper bound for the number of observations is reached. In our case, this is reached when the upper limit of blocks have been administered. Additional modifications include conducting two-stage estimation to allow sufficient observation for preliminary estimates of the voxel-level error variance from a first stage where stopping is not yet considered (Hall, 1962). With these estimates, we can derive an alternative hypothesis value for a linear contrast of task parameters *cβ* that will correspond to a desired *z*-statistic value, denoted as *z_t_*. As an illustration, suppose a *z*-statistic threshold value of 3.10 is selected, as will be done below in our studies. (Note *z_t_* = 3.10 is associated with the one-sided *p*-value = 0.001). Given an estimated value $\hat{var}\left[c\hat{\beta_{t}}\right]$ from a first stage of length *t* scans, we solve for the value of θ_1_ = *cβ*_1_ that satisfies:


(1.6) $$\frac{\theta_{1}}{\sqrt{\hat{var}\left[c\hat{\beta_{t}}\right]}}=z_{t}$$

This value becomes the alternative hypothesis, and it represents the voxel-level targeted activation magnitude threshold. For subsequent scans we fix the value of θ_1_ as the alternative hypothesis value for *cβ*, so that the activation magnitude threshold in the alternative hypothesis is held constant. Note that *z*_*t*_ will thus tend to increase across scans, since $\hat{var}\left[c\hat{\beta_{t}}\right]$ decreases with more data. This approach allows for more comparable activation patterns across different scan lengths, since respective activations reflect similar magnitudes.

Ultimately, we aggregate the findings of the voxel-level SPRTs to determine whether or not experimentation within a block design should be terminated early. A “global” stopping rule that considers all voxels in a region of interest (can be whole brain or smaller ROIs) that we have adopted is to terminate task administration when a predetermined percentage of voxels have been classified by their respective SPRTs. For instance, we have used 80% as a global stopping criterion. Note that 80% classified means either as active or non-active. We choose this cut-off as it is fairly strict, and yet approximately one half of the participants still stop early. As we will see, it also facilitates correspondence with full scan data results, particularly if the activation threshold is adjusted to recognize longer scan durations. We also consider other global stopping criterion here, 70 and 90%, and assess impact on stopping times and resultant images arising from early stopping. We also choose the SPRT target Type I and Type II error levels that are relatively more stringent for Type I error. Note that for *cβ* parameter values that are “in-between” the null and alternative hypothesis values, the SPRT is indifferent to preferring one hypothesis over the other. This leads to larger numbers of scans needed before a stopping boundary is crossed. So, we have to accept a lack of decisive stopping decisions for these cases in order for overall experimentation to stop early. This can be an acceptable trade-off for shorter experimental scan times and the ability to tailor experimentation.

## Methodology

### Participants

Twelve healthy subjects were recruited, 7 males. They were aged 15–16 years old and 11 were right-handed. They had no known neurological conditions and a normal developmental history. A group of 11 adolescents born EPT were also recruited, 1 male. EPT was defined as being born at <26-week gestation and weighing < 1000 g. All were aged 15–17 years old and 8 were right-handed, 2 left-handed and 1 ambidextrous. All subjects were recruited as part of a larger study to evaluate functional and structural differences associated with mathematical abilities and working memory between those born EPT and those born at normal term. The aim of the larger study is to improve our understanding of mathematics disabilities and potentially lead to improvements in pedagogical practices for young people experiencing problems acquiring mathematics skills. Adolescents were recruited as they can handle the stress of fMRI experimentation, are mathematically advanced enough and have had time to master the subject area compared to younger children. This age range is also an advantageous time to implement interventions to improve mathematical abilities before leaving school, hence adults were not studied. EPT subjects were included to show that differences with patient populations are detectable with our methods. A subsection of the full study is reported here to demonstrate the real-time analysis.

The subjects made one two-h visit to the MRI department at University Hospitals Cleveland Medical Center (UHCMC). Ethics approval was obtained from the UHCMC Institutional Review Board office prior to the study and complied with the Declaration of Helsinki for human subject research. Subjects and their parents gave informed consent prior to taking part.

As part of our wider study, subjects also made another, separate 3-h visit to the study offices to undergo neuropsychological testing and a refresher of fraction calculations. In the interests of brevity, the full neuropsychological testing results are not reported here. One finding that is particularly relevant to the fMRI task considered here is that nearly two thirds (63.6%) of the EPT cohort have lower working memory function, compared to just over one third (35.7%) of controls subjects. Working memory level was determined by sample quartiles of standardized z-scores based on the Wide Range Assessment of Memory and Learning test (WRMAL^1^, Sheslow and Adams, 2003). We denote “lower” working memory function as having a standardized score that is less than the sample median.

### MRI Protocols

The subjects were positioned head-first supine on the scanner bed with their head fixed in position using inflatable pads. An 8-channel head coil was used for data acquisition. Echo planar imaging scans were acquired on a Philips Ingenuity 3T PET/MR imager at UHCMC. The following fMRI scan parameters were used: TR = 3.0 s, TE = 35 ms, in-plane resolution was 1.797 mm^2^ (matrix 128 × 128), slice thickness was 4 mm, number of slices = 36 slices and flip angle = 90°. A SENSE P reduction factor of 2 was implemented and scans were acquired in an ascending interleaved fashion.

In addition to the fMRI scans, a high-resolution T1-weighted anatomical image of the brain was also acquired. This was taken using a magnetic preparation gradient-echo sequence (3D IR TFE). Imaging parameters were: TR = 7.5 ms, TE = 3.7 ms, in-plane resolution was 1 mm^2^ (matrix 256 × 256), slice thickness was 1 mm, number of slices = 200 slices and flip angle = 8°.

### Stimulus Protocols

**FIGURE 1:**
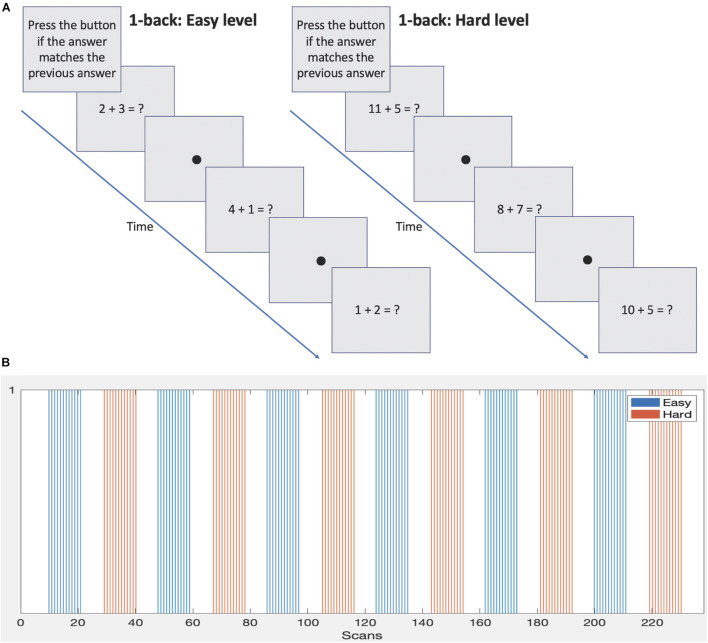
**(A)** Sample 1-back protocols demonstrating the two difficulty levels. **(B)** Block design and timings of each difficulty level.

During data acquisition subjects were presented with a mathematical version of the well-known 1-back memory task. It involved performing basic addition and subtraction calculations and required the answer to be remembered and compared to the next answer. Two difficulty levels were included. The protocol was developed by our lab as part of a battery to assess mathematical and working memory abilities in 14–17 year olds to evaluate the functional differences between those born EPT and those born at normal term. The stimulus was presented on an MRI compatible LCD monitor (manufactured by Cambridge Research Systems, Rochester, United Kingdom) positioned at the end of the bore and viewed via a mirror attached to the head coil. Equations were presented, for example, the subject may see “2 + 3 = ?.” The subject was required to work out the answer and then remember it while working out the next equation, for example “1 + 4 = ?.” If they thought the answers matched, then the subject pressed a button on a response box held in their right hand. If they thought the answers did not match, then they did nothing but remember the new answer to compare to the answer of the next equation. An example sequence is shown in Figure 1A.

The stimulus was presented in a block design, see Figure 1B and Table 1. Eight equations were presented per block. Each block lasted 36 s followed by 21 s of rest condition (fixation dot). Two difficulty levels were presented. The easier level consisted of single digit numbers to add or subtract and the answers were always a single digit. The harder level involved addition or subtraction of single or two-digit numbers and the answers were always two digits. Blocks of difficulty levels were alternated during the scan and a total of 6 blocks per level were presented. Note: although only 2 difficulty levels are used here, the setup is able to accommodate any number of difficulty levels. The full duration of the task was 238 scans or 11 min and 54 s. This was based on a moderate length of experimentation for a 1-back block design (e.g., see Daamen et al., 2015; Poudel et al., 2015; Di et al., 2020; Huang et al., 2020), allowing approximately 6 min for each difficulty level.

**TABLE 1:** The scan number when each stimulus block is completed.

Block	Easy level	Hard level
1	21	40
2	59	78
3	97	116
4	135	154
5	173	192
6	211	230

Two difficulty levels were included to investigate differences in neural responses associated with increasing task demand. As the brain is ‘pushed’ to solve more complex problems, differential networks may be apparent, and these may be different between normal term and EPT subjects. Additionally, increasing the difficulty level serves to maintain the subject’s attention and, generally, increases their effort. This can have the effect of increasing brain activation cluster sizes and magnitude as well as causing recruitment of additional areas, which is of interest. Incorporating difficulty levels into protocols that can separately be terminated early demonstrates the flexibility of the proposed approach.

### Real-Time Functional Magnetic Resonance Imaging Acquisition

The visual stimulus was presented using an in-house custom written program that was developed using the Python programming language (Python Software Foundation^2^) and libraries from PsychoPy - an open source visual presentation program (Peirce, 2007, 2009; Peirce and MacAskill, 2018). The program connected to a Cedrus Lumina controller to receive stimulus responses from the subject and trigger pulses from the MRI scanner (outputted every dynamic). The timing of the presentation of the visual stimulus was synchronized to the trigger pulses to ensure that stimulus images were displayed at the expected time. A Supervisor Window displayed on the experimenter’s computer screen allowed the visual stimulus to be tracked throughout. It displayed the current block number being presented, how many remaining blocks there were and when the subject responded. The program was also able to terminate one or both of the difficulty levels if it received a signal indicating the relevant areas in the fMRI data were sufficiently classified across voxels. The program offered the flexibility to automatically terminate the presentation or the experimenter could override the termination instruction and continue stimulus presentation. The software is freely available from the Bitbucket repository^3^.

Real-time image transfer was achieved by XTC (eXTernal Control). This is a program integrated into the Philips scanner software and enabled by a research clinical science key. XTC communicates with the reconstruction and scanner processes on the scanner computer and interfaces to a network client application using a minimalistic CORBA (Common Object Request Broker Architecture) (Schmidt et al., 1998) interface which uses TCP/IP as the transport layer. CORBA is platform independent, reliable, and has the ability to process large amounts of data with minimum overhead. Each CORBA message consisted of a hierarchical attribute collection identified with UUIDs (universally unique identifiers) (ITU-T, 2005). Messages carried reconstructed image data and meta-data containing details of scan protocols. Due to hospital network security protocols the reconstructed images were placed in a folder on the scanner computer and then pushed across to a Linux computer. To achieve necessary image transfer speeds to the scanner computer folder a modification to XTC was installed on the scanner to disable two-way communications as only one-way image transfer functionality was required. However, XTC does support two-way communication between the scanner and the Client.

**FIGURE 2:**
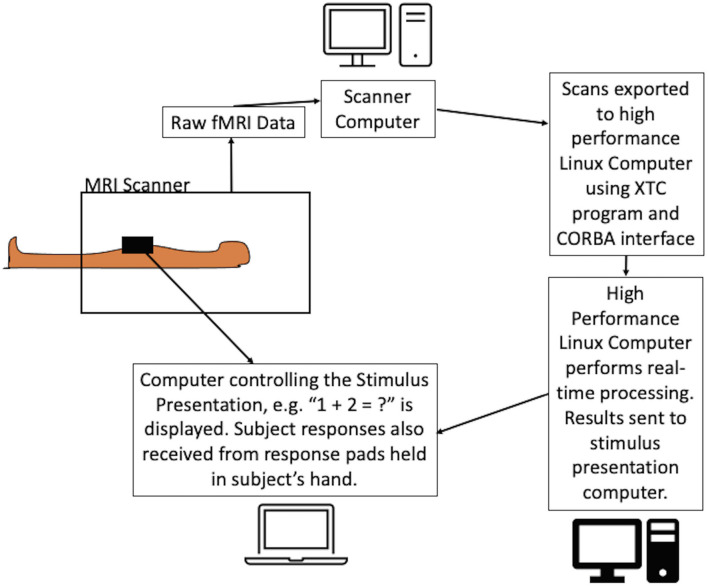
Schematic of the experimental setup of the dynamic real-time fMRI process. The equations were presented to the subject while the scans were acquired using a dedicated computer. FMRI scans were exported in real-time from the scanner computer to the Linux workstation using the Philips XTC program and CORBA interface. Scans were preprocessed on the Linux workstation and SPRT statistics were calculated. The results were relayed back to the stimulus presentation program with an instruction to either continue or terminate the stimulus. The program allowed the flexibility to automatically terminate the presentation of the difficulty level or it could be overridden by the experimenter to continue presenting the stimulus. Note: where hospital firewalls are not an issue, the setup can be simplified.

The Linux computer was a custom-built server equipped with a solid state hard drive and two 8-core Intel Xeon E5-2687W processors running at 3.1 GHz and providing 40 MB L3 cache. It was installed with Centos 7.4 operating system. As the scans were received, custom written Python and Bash scripts implemented the analysis using core-based parallelization to preprocess the data and perform the SPRT statistical analysis. Preprocessing was performed using standard modules from AFNI (Analysis of Functional NeuroImages^4^) and FSL (FMRIB’s Software Library^5^). The analysis sequence is detailed in the following section. The setup is shown in Figure 2.

### MRI Preprocessing

At the beginning of the scanning session a single fMRI scan (3 s) was acquired and used for coregistration (motion correction) purposes. In preparation, the skull was removed using FSL’s Brain Extraction Tool (BET) (Smith, 2002) and a mask of the full brain was created. During the real-time adaptive fMRI scan session, new scans arrived every 3 s and were dumped in a folder on the Linux workstation where the following actions were applied to each one. AFNI’s ‘dcm2niix_afni’ command was used to convert the .par/.rec files to NIfTI. Motion correction was performed using coregistration techniques. Every fMRI scan was realigned to the initial scan that was acquired before the task began, and AFNI’s ‘3dvolreg’ command was used. Spatial smoothing was also applied using an 8 mm kernel with AFNI’s ‘3dmerge’ command. The full brain mask created at the beginning of the session was applied using FSL’s ‘fslmaths’ command to remove noisy voxels outside the brain (voxels of no interest). CSF and white matter regions were included in the full brain mask. This was due to time constraints at the beginning of the scanning session that did not allow for segmentation of the brain. However, it is feasible to create a ROI (full brain/segmented brain/particular structure) prior to the start of the scan session. If a pre-existing scan of the subject is not available for this purpose then the ROI can be created using a template brain (e.g., MNI) and morphed to the subject’s initial single fMRI scan. This process was tested as a part of our study and is feasible to conduct, although not used here.

The resulting preprocessed images were then converted to ASCII text files for statistical analysis with SPRT producing one .txt file per fMRI scan. The text file contained the grayscale values for every voxel in a 3D image. The conversion was accomplished using ‘fsl2ascii’ command.

### Functional Magnetic Resonance Imaging Sequential Probability Ratio Test Analysis

The SPRT analysis was applied using a highly optimized C++ program that used Intel Cilk Plus library for multicore and vector processing of data. BLAS routines from Intel MKL were used to enable instruction-based acceleration for matrix computation. They are available from the Bitbucket repository at https://bitbucket.org/tatsuoka-lab.

The design matrix was created prior to the scan session and contained 8 columns. One column of ‘1s’ is for the intercept. Two columns represented the easy and hard stimuli. They were created using AFNI’s ‘3dDeconvolve’ command to model the HRF. Easy and hard levels were modeled separately and the contrasts applied post processing were easy or hard versus rest. We also included in the design matrix cosine functions of increasing periodicity to model major sources of noise in fMRI data. These include brain metabolism, physiology, and spontaneous fluctuations (Friston et al., 2000; Yan et al., 2009). For large periodicities, cosine functions are approximately linear for the time frame of scans we consider here, and hence are essentially collinear from a GLM modeling perspective. Five regressors were thus added to the design matrix and were formulated based on the information given in Kiebel and Holmes (2006). The frequencies were based on total/maximum scan duration and were: maximum scan duration^∗^2, maximum scan duration, maximum scan duration/1.5, maximum scan duration/2 and maximum scan duration/2.5. It is also possible to include the temporal derivatives of the HRF or other regressors in the design matrix where applicable in studies. Temporal derivatives were not included here due to the long durations of the block design used to present the task. Motion parameters are also frequently used as regressors to remove correlated activations produced by movement. Here motion parameter regressors were not included with the estimation of the discrete cosine transforms due to the limitations of the computational resources.

Important design parameters for implementation were selected through analysis of a training sample. For training, each subject underwent the full duration of experimentation. We determined design parameters through the following criteria:

(1) First stage duration: It is desirable for the voxel-level error variance and beta parameter estimates to stabilize – we assessed this qualitatively by assessing plots from a sample of voxels. We tested 2 scenarios using either 2-blocks or 4-blocks of easy and hard stimuli first stage administration before allowing early stopping to occur. Where 2-blocks per difficulty level of stimulus administration were used before allowing early stopping, the first 78 scans were used for the first stage of experimentation. Where 4-blocks were used, 154 scans were used for the first stage. Recall, a full-length task protocol comprised 238 scans and lasted 11 min and 54 s.

(2) Activation threshold: We chose a *z*-score threshold of *z*_*t*_ = 3.10 after the first stage since this is a standard threshold value for determining activation of a voxel. We then solved for θ_1_, as in (1.6). After this first stage, the null and alternative hypotheses at a given voxel are set as *H*_0_: *cβ* = 0 and *H_a_: cβ* = θ_1_.

(3) Target Type I and Type II error levels: Typical values used in the literature were used to test stopping time performance, with α_E_ = 0.001, *β*_E_ = 0.1 (Banerjee et al., 2009; Cremers et al., 2017). We also considered α_E_ = 0.0001, *β*_E_ = 0.1 and α_E_ = 0.001, *β*_E_ = 0.01 combinations as well.

(4) Global stopping percentage: We investigate the scenarios of classifying 70, 80, and 90% of voxels. Ideally, early stopping occurs while there is still correspondence in activation patterns to full scan durations, and general concurrence with expected activations from neuroscientific literature.

The stopping rules were defined as described in Section “Sequential Probability Ratio Test”:

1. Continue sampling, if *B* < Λ_*t*_ < *A*
2. Stop sampling and accept *H*_0_, if Λ_*t*_ < *B*
3. Stop sampling and accept *H_a_*, if *A* < Λ_*t*_

where stopping boundaries are given in (1.4) and are based on target Type I and II error levels. The error levels are specified before testing begins.

Although our goal was to stop scanning early it was necessary to acquire first full length datasets to fully verify our analysis methods and check the feasibility of stopping early. In addition to the subjects reported in the results, we tested early stopping in 2 other subjects to confirm that our procedures worked. Subject details and data are not reported for them as full datasets are not available.

This 1-back arithmetic task involves not only number sense and mathematical calculations but also general cognitive skills involving working memory and sustained attention. The brain networks involved with each of these have been well characterized in the literature and lends itself to the evaluation of this real-time analysis method. There is a large amount of overlap for the active brain areas that control each of these functions and they appear as a frontoparietal network (Menon, 2016a, b; Chai et al., 2018). The areas of the brain we expect to activate in response to each contrast of easy versus rest and hard versus rest are: the intraparietal sulcus, supramarginal gyrus, premotor cortex, dorsal/ventral lateral prefrontal cortex, parietal lobe, Broca’s area, occipital lobe, fusiform gyrus, precuneus, cingulate gyrus, anterior insula and frontal eye fields. Assessment of the location and extent of activations within this network will be used as additional criteria for judging appropriate stopping times, in addition to the statistical information determined through the SPRT analysis. This will include how well the cluster peaks coincide with the anatomical locations as well as their extent.

### Group Analysis

There are many possible applications in the research setting where individual level results may be the focus. A possible clinical application may be in clinical assessments for presurgical evaluation for brain surgery in patients with brain cancer or epilepsy. Still, group analyses are commonly conducted and form an essential aspect of fMRI analyses. The outputted results files from the SPRT analysis can be used directly to perform a group analysis using AFNI’s 3dMEMA command (Mixed Effects Meta Analysis tool) (Chen et al., 2012), even with the variable scan durations. However, a group analysis was carried out using FSL which instead merges all subject data to conduct a combined mixed models analysis. We demonstrate that the data collected in real-time can still be used in a typical *post-hoc* analysis and can be processed with different parameters to those specified in SPRT. Raw data was preprocessed with FSL FEAT (Woolrich et al., 2001). Motion correction was performed using a rigid body transform, spatial smoothing with a full-width-at-half-maximum Gaussian kernel of 6 mm was applied, high pass temporal filtering of 90 s was carried out and coregistration to (MNI) standard space was done before performing a first level individual GLM analysis. The statistical output from these were used to perform the higher level group statistics using FLAME 1 [FMRIB’s Local Analysis of Mixed Effects, (Woolrich et al., 2004)].

## Results

### Individual Subject Results of Sequential Probability Ratio Test

The median control subject response time across both difficulty levels was 1.44 s (SD 0.51 s), and median task accuracy was 90.8% (SD 20.2%). When these are broken down by difficulty level, the easy level median task accuracy was 86.1% (SD 22.6%) with median response time of 1.28 s (SD 0.54 s); and the hard level median task accuracy was 90.0% (SD 18.4%) with median response time of 1.56 s (SD 0.51 s). EPT subjects had a slightly longer overall median response time of 1.91 s (SD 0.48 s) and overall median task accuracy was lower at 65.8% (SD 21.2%). For the easy level, the median accuracy was 72.2% (SD 24.2%) and median response time was 1.63 s (SD 0.49 s). For the hard level the median accuracy was 70.0% (SD 19.8%) with a median response time of 2.10 s (SD 0.54 s). Note that there are statistically significant differences in same subject differences in speed to completion by difficulty level (Wilcoxon signed rank test, two-sided *p* < 0.001). Comparing correctness percentages per subject across birth status groups, there are significant differences with the hard level (Mann-Whitney *U*-test two-sided *p* = 0.037), but not with the easy one (two-sided *p* = 0.401). These results indicate that the difficulty levels have different psychometric properties, and affect the groups differently. We also see this in activation patterns, as discussed in Section “Group Analysis Results” and reflected in the group analysis results.

Real-time transfer speeds between the scanner and the single Linux workstation were consistently fast, with individual scan files taking less than 150 milliseconds to transfer. All subject scans were processed within the 3 s TR period. Offline testing showed that the subject with the largest number of voxels (subject 21 with 135,379 voxels) required 145 s to conduct all SPRT analyses, starting from scan 79. Less than 1 s is required to analyze the first stage data. The subject with the fewest number of voxels (subject 14 with 77,359 voxels) required 79 s for SPRT analyses across the scans. Overall real-time processing times in this workflow were due in most part to pre-processing computations. For the subject with the largest number of voxels, the maximum time to process one scan in real-time would be about 1.6 s (0.54 s preprocessing time + 0.91 s SPRT calculations + 0.15 s transfer time). This would be even faster with more computational resources, faster transfer speeds or reducing the number of voxels being analyzed.

Inspection of the *z*-score maps for easy and hard levels for each subject showed that generally, across subjects, the largest activations were centered bilaterally around the inferior and superior parietal areas, taking in the intraparietal sulcus, a region highly associated with mathematical functioning. Further activations were seen in the cuneus. These are most likely correlated with the visual processing associated with the task. Additional activations were seen in the precuneus, bilateral areas in the medial frontal gyrus, anterior cingulate, insula and inferior frontal gyrus. These areas are often associated with attention and memory systems (Cavanna and Trimble, 2006; Menon, 2016a).

**FIGURE 3:**
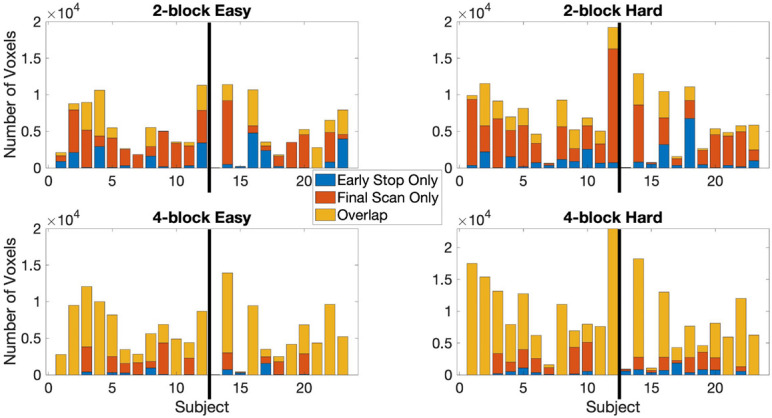
The active voxel counts shown are for the voxels-in-common between the full duration scan and early stop scan and counts are for active voxels unique to either the final scan or early stop scan. Top row: 2-block easy and hard levels, bottom row: 4-block easy and hard levels. Maximum number of possible scans is 238, minimum is 78 scans for 2 blocks first stage of easy and hard stimulus administration or 154 scans for 4 blocks first stage of easy and hard. Information given for the point where 80% of voxels have been classified as either active or non-active. Results reported uses α_*E*_ = 0.001, *β*_*E*_ = 0.1, and *z* = 3.10 (*p* < 0.001) at first scan after the first stage. The thick black line in each chart indicates the subject groups. Left of the black line (1–12) = control subjects, right of the black line (13–23) = EPT subjects.

The stopping times for the 2- and 4-block first stage lengths for various criteria for each subject are given in Table 2. The spatial overlap between early stopping and full duration maps are explored in Figure 3 where we show the number of active voxels in common between the two durations. The median spatial overlap where early stopping occurs for control subjects was 20.0% (SD 27.0%) for the easy level, 2-blocks and 76.3% (SD 34.5%) for easy level, 4-blocks. For the hard level, there was 30.7% (SD 16.4%) and 77.2% (SD 26.5%) overlap for the 2-block and 4-block first stages, respectively. For EPT subjects the median overlap was 28.7% (SD 35.6%) and 82.7% (SD 34.5%) for the easy level, 2- and 4-block first stages, respectively. For the hard level, the median overlap values were 21.3% (SD 20.6%) and 82.5% (SD 26.5%) for 2-block and 4-block first stages. In Supplementary Table 1, maps of activations for instances where there is less than 50% overlap are presented.

**TABLE 2:** Subject early stop durations of the 1-back task using SPRT.

Subject	Scan when 80% Reached 2-Block Easy	Scan when 80% Reached 2-Block Hard	Scan when 80% Reached 4-Block Easy	Scan when 80% Reached 4-Block Hard
**Control**				
1	4E/3H	2E/2H	Not reached	Not reached
2	3E/2H	3E/3H	Not reached	Not reached
3	3E/2H	2E/2H	5E/4H	5E/4H
4	4E/3H	3E/2H	Not reached	5E/5H
5	3E/2H	2E/2H	5E/4H	5E/4H
6	2E/2H	3E/2H	5E/5H	6E/5H
7	2E/2H	3E/2H	5E/4H	4E/4H
8	3E/3H	3E/2H	5E/5H	Not reached
9	2E/2H	3E/2H	4E/4H	4E/4H
10	3E/3H	2E/2H	Not reached	4E/4H
11	3E/2H	3E/3H	5E/4H	Not reached
12	3E/3H	3E/2H	Not reached	Not reached
EPT	2E/2H	2E/2H	4E/4H	4E/4H
13	3E/3H	2E/2H	5E/5H	6E/6H
14	3E/3H	3E/2H	4E/4H	5E/4H
15	3E/3H	3E/2H	Not reached	5E/5H
16	2E/2H	2E/2H	5E/4H	6E/5H
17	2E/2H	4E/3H	4E/4H	6E/6H
18	3E/2H	2E/2H	Not reached	4E/4H
19	2E/2H	2E/2H	4E/4H	4E/4H
20	Not reached	2E/2H	Not reached	Not reached
21	3E/3H	3E/2H	Not reached	6E/6H
22	3E/3H	3E/2H	Not reached	Not reached
23	4E/3H	2E/2H	Not reached	Not reached

*Analysis reported here uses α_E_ = 0.001, β_E_ = 0.1, and z = 3.10 threshold at first scan after the first stage. Maximum number of possible scans is 238, minimum is 78 scans for 2 blocks first stage of easy and hard stimulus administration or 154 scans for 4 blocks first stage of easy and hard. Information given for the point where 80% of voxels have been classified as either active or non-active. E = administration of easy level block, H = administration of hard level block.*

**FIGURE 4:**
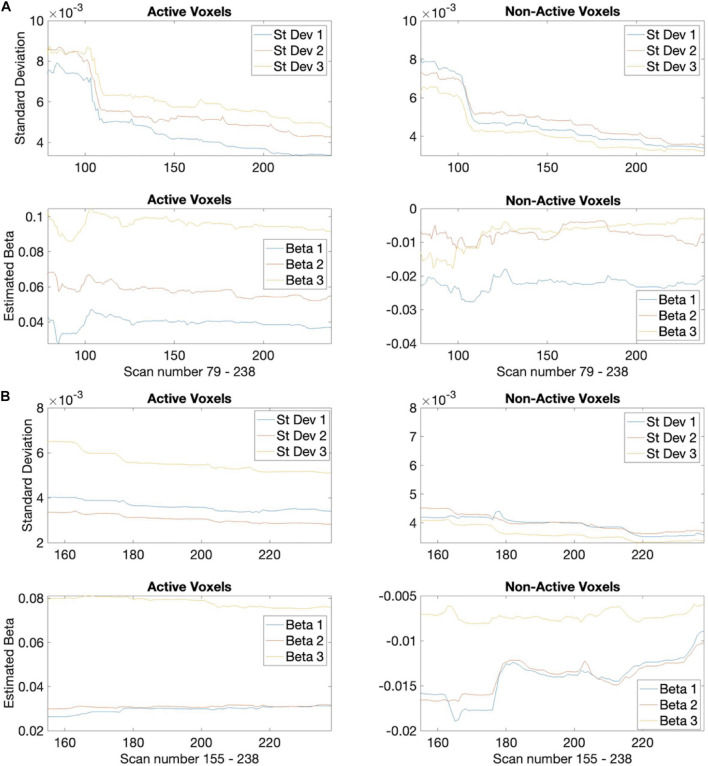
Estimated standard deviations for the easy task parameter. Plots for 3 example active (left) and non-active (right) voxels from a control subject (subject 3) showing how the estimates decrease over time (scan number). **(A)** Based on 2-block first stage estimation, **(B)** 4-block first stage estimation.

**FIGURE 5:**
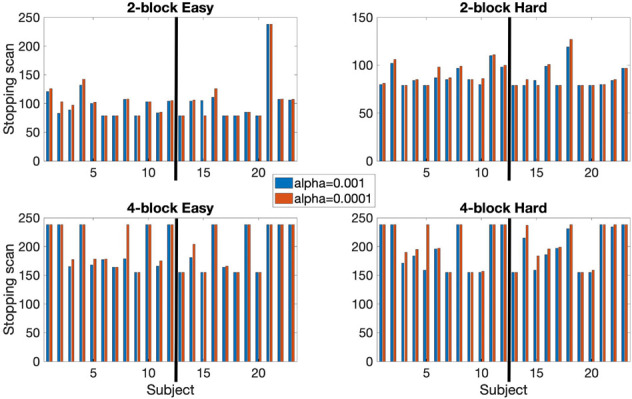
Comparison of early stopping times using α_*E*_ = 0.001 and α_*E*_ = 0.0001. Based on 80% of voxels being classified. Both 2-block (top row) and 4-block (bottom row) first stage conditions are presented. *β*_*E*_ = 0.1 throughout. The thick black line in each chart indicates the subject groups. Left of the black line (1–12) = control subjects, right of the black line (13–23) = EPT subjects.

The median spatial overlap for the 2-block first stage is less than 50% for both control and EPT groups. We see for instance in Figure 4, error variance and beta estimates are generally not stable after a 2-block first stage. It is important to “wait” until this happens, as these parameters play a central role in inference and on test statistic values. The 4-block first stage is more attractive in this way. Figure 5 indicates how early stopping is affected by the SPRT Type I error threshold values. Type II error levels seemed to have higher impact. For *β*_*E*_ = 0.01, although not shown, early stopping appears somewhat less often and closer to the end of the experiment.

Stopping was reached at 80% of voxels classified as either active or non-active in all subjects with the exception of one instance (EPT subject, easy level) for 2-block first stage. For 4-block first stage, 7/12 control subjects stopped early for both the easy and hard levels. Among the EPT group, 6/11 subjects stopped early for the easy level and 9/11 subjects stopped early for the hard level. The median stopping duration for both difficulty levels for control subjects was 3 blocks of easy and 2 blocks of hard stimulus administration for 2-blocks first stage. For 4-blocks first stage, the median stopping time was 5 easy, 4 hard for both difficulty levels. In EPT subjects, the median stopping time for 2-block first stage was 3 easy, 2 hard blocks of stimuli. For 4-blocks first stage, the median stopping time was 4 easy and 4 hard for the easy level and 5 easy, 5 hard for the hard level. Depending on the number of first stage blocks, time savings of 1/3 to 2/3 (4 to 8 min on a 12-min scan) can be achieved.

**FIGURE 6:**
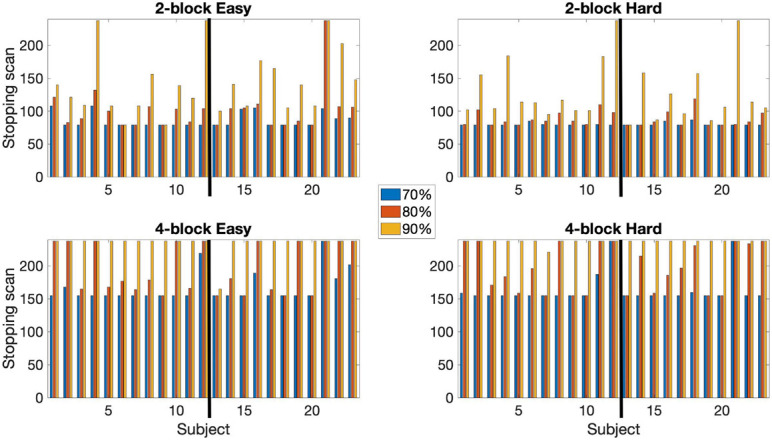
A comparison of the early stopping scans at 70, 80, and 90% of voxels classified as either active or non-active. Conducted using α_*E*_ = 0.001, *β*_*E*_ = 0.1. Both 2-block (top row) and 4-block (bottom row) first stage conditions are presented. The thick black line in each chart indicates the subject groups. Left of the black line (1–12) = control subjects, right of the black line (13–23) = EPT subjects.

An early stopping rule based on a classification rate of at least 70 or 90% was also tested. Results reported in Figure 6. At 70% classification most subjects stopped early. For the 2-block first stage all subjects stopped early. Median stopping scan number was 79 for both the easy and hard levels for both groups. For the 4-block first stage – easy level, 1 EPT subject did not stop early, and 1 EPT and 1 control subject did not stop early for the hard level. Median stopping scan was 155 for both difficulty levels for both groups. At 90% classification, most subjects stopped early with 2-block first stage but most did not stop early with 4-block first stage. For the 2-blocks first stage condition, 3 subjects did not stop early for the easy level and 2 subjects for the hard level. Only 1 subject stopped early under the 4-blocks first stage condition for the easy level and the hard level. When analyzing counts of voxels classified as active or non-active between these rules, the 80% thresholds lead to more non-active classifications, but the difference in active voxels is less systematic. Given that early stopping occurs almost invariably with the 70% rule, this value should also be considered. Supplementary Table 2 provides plots of the percentage of voxels that are, respectively, classified as active and non-active over the course of the full scanning duration based on 80% of voxels being classified. A general trend is that the percentage of non-active voxels gradually decreases while that of active voxels increases. Longer scan durations also allow for some adjustment in *post-hoc* analyses, and may have some potential advantages for group analysis, as discussed below. Hence, we present results for the more conservative 80% rule, which leads to relatively longer durations even when early stopping occurs.

In summary, these results indicate that early stopping is feasible but that the parameters need to be carefully chosen. The desire for earliest possible stopping must be balanced with selections of a first stage estimation duration and a voxel classification percentage which help insure that the number of active voxels will significantly overlap with those from a full scan, and that a sufficient amount of experimentation has been conducted. Of the parameters we tested, our results indicate that useful results were obtained using 4-blocks first stage with 80% classification. We discuss this further in the Discussion section.

**FIGURE 7:**
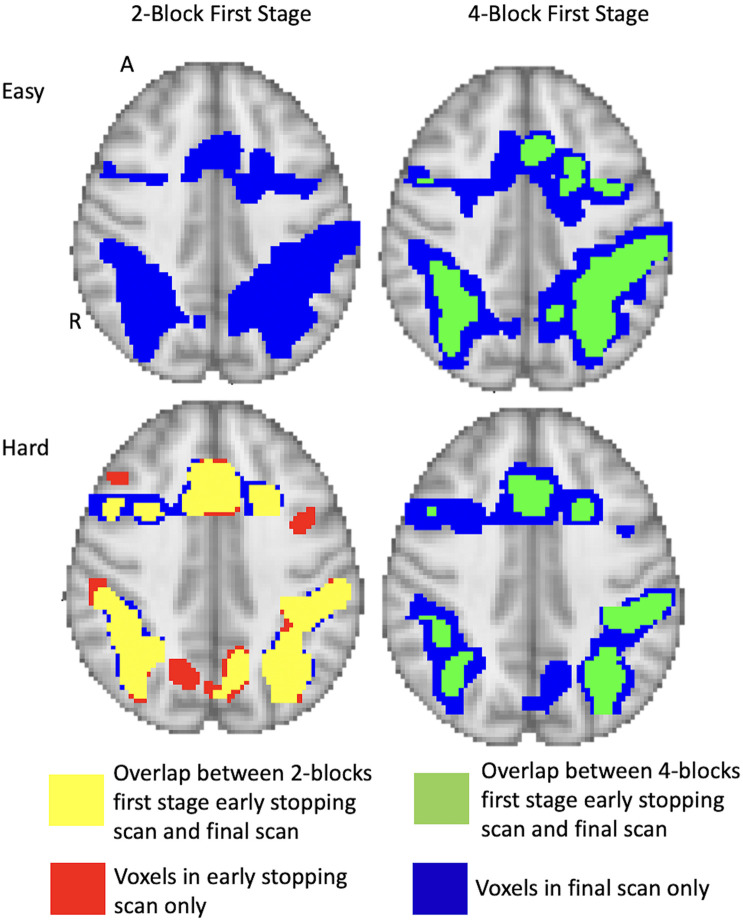
Full brain activation maps showing the overlapping voxels between the different stopping points (using 2-blocks first stage, 4-blocks first stage and final scan). Top row shows the easy level and bottom row shows the hard level for 1 subject (number 9). The voxels that are active only at full duration are shown in blue. Those only active after 2-blocks or 4-blocks of stimulus administration are in red. Yellow shows the overlap between full duration and 2-block first stage early stopping scans. Green shows the overlap between full duration and 4-block first stage stopping scans. Thresholded at *z* = 3.10 at first scan after the first stage. Results overlaid on MNI template, slice *z* = 56 shown. R = right, A = anterior.

The activation maps under the different conditions are shown in Figure 7 for a sample subject (subject 9). For the 2-block first stage, this subject terminated after 2 blocks of easy and hard administration for the easy level (scan 79) and after 3 blocks of easy and 2 blocks of hard administration for the hard level (scan 85). For the 4-block minimum, this subject terminated at scan 155, equal to 4 blocks of easy and hard stimulus administration, for both difficulty levels. The images show that at scan 79 there is very little activity present and the majority of the voxel classifications are non-active. By scan 155, there is much more activity which has a similar pattern to the final scan. The extent is not quite as large as the final scan, however, the foci of the clusters do overlap showing good correspondence between the two different time points. Other subject activation maps for instances where there is less than 50% overlap are shown in Supplementary Table 1.

By holding the alternative hypothesis threshold θ_1_constant after the first stage, note that the corresponding *z*_*t*_-score associated with the θ_1_threshold actually increases as the scan durations increase. The decreasing variance of $c\hat{\beta_{t}}$ is approximately proportional to *c*(*X′X*)^–1^*c*′ (as it would be under constant error variance and no serial correlation). Hence, for the easy level task, a *z*_*t*_-score of 3.1 for 154 scans approximately corresponds to a *z*_*t*_-score of 4.0 for the final scan (238 scans), holding θ_1_ fixed. At 78 scans, a *z*_*t*_-score of 3.1 corresponds to a *z*_*t*_-score of approximately 8.37 at 238 scans, so there may be less overlap between early stopping and full duration for shorter first stage durations. See Supplementary Table 1 for images resulting from different stop rules and first stage durations. In Supplementary Table 2, the trends in percentage of voxels classified as active and non-active reflect this phenomenon, at least for some of the subjects.

Importantly, the issue of whether early stopping or full duration provide better activation maps is best answered neuro-scientifically, through the support of literature and hypotheses. We used this training sample to assess whether early stopping activation patterns were reasonable, or too early, and compared them with full duration results. In the Supplementary, we add plots for early stopping versus full duration for each subject for which early stopping was invoked. Comparing these plots, we see that in many instances that the cluster peaks are located in the expected anatomical locations.

In summary, although there are similar rates of early termination between the 2-block and 4-block first stage cases, the detected activation patterns suggest that using 4-blocks of stimulus administration is more suited to determining active voxels. In Figure 4, to illustrate the rate of decrease of the estimated $\hat{var}\left[c\hat{\beta_{t}}\right]$ values, we present a plot of $\hat{var}\left[c\hat{\beta_{t}}\right]$values for one subject across a set of voxels over the duration of the experiment. These values give an indication that stopping based on the θ_1_-values at the end of the 2-block first stage may be too early for correspondence with full duration scans, as the estimated standard deviations are relatively larger and the corresponding θ_1_-values for *z* = 3.10 in the alternative hypothesis can be larger as well, compared to a 4-block first stage.

**FIGURE 8:**
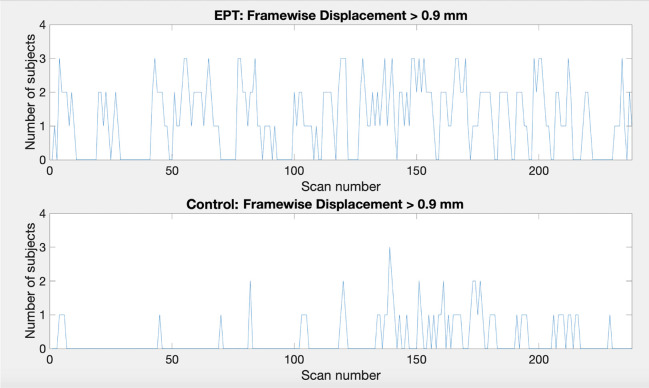
Plots showing the number of subjects that pass the framewise displacement threshold of 0.9 mm for each scan. Top: EPT subjects, bottom: control subjects.

Note that in a subset of subjects there was some volatility due to subject movement. This may in part explain why sometimes there is a lower number of active voxels for the easy task for full duration than when stopping early. In most subjects the active voxel count increases with scan duration. Subjects 13, 15 and 17 had the most pronounced decrease of active voxel counts. Subject 13 demonstrates very few active voxels at all and there is almost no consistency in location. Further investigation shows large relative framewise displacement occurs frequently throughout the scan and many of the responses have been missed or have relatively long response times, 60.5% correct overall and 2.08 s (SD 0.97 s) average response time (see plots for subject 13 in Supplementary Table 3). Taken together these suggest that either the task level may not have been aimed at the right level and/or the subject may have been uncomfortable and distracted in the scanner thereby attending to the task less than required for robust activations to occur. Subject 15 demonstrates consistent clusters but their size decreases over time. Framewise displacement shows very little motion, particularly from scan 180 onwards. The response plots (in Supplementary Table 3) show the subject is paying attention and responding appropriately. Subject 17 has a similar pattern of decreasing cluster sizes. The framewise displacement plots indicate a moderate amount of motion throughout. Although the subject has missed many of the task questions (65.8% correct), the pattern of responding indicates they are awake and attending to the task. In general, EPT subjects demonstrated more motion. The median number of scans with framewise displacement above a threshold of 0.9 mm [threshold determined from Siegel et al. (2014)] was 5 scans (SD 43 scans) for EPT subjects and 2.5 scans (SD 8 scans) for control subjects. One EPT subject passed the threshold a total of 124 scans out of 238 scans. In contrast, the control subject with the maximum number of threshold passes was 30/238 scans. This is further demonstrated in Figure 8 where we show subject counts for each scan when the threshold has been passed. For both EPT and control subjects, it is clear that subjects are moving more frequently in the second half of the scans and supports stopping early to reduce motion artifacts and noise in the data. Formally, we see statistically significant differences when comparing counts of motion events with framewise displacement greater than 0.9 mm in the first versus second half of scanning (*p* = 0.003, two-sided signed rank test). EPT group also has significantly more movement in the first half of scanning (*p* = 0.035, two-sided Mann-Whitney *U*-test), indicating a group-level proclivity for more motion events.

### Group Analysis Results

**FIGURE 9:**
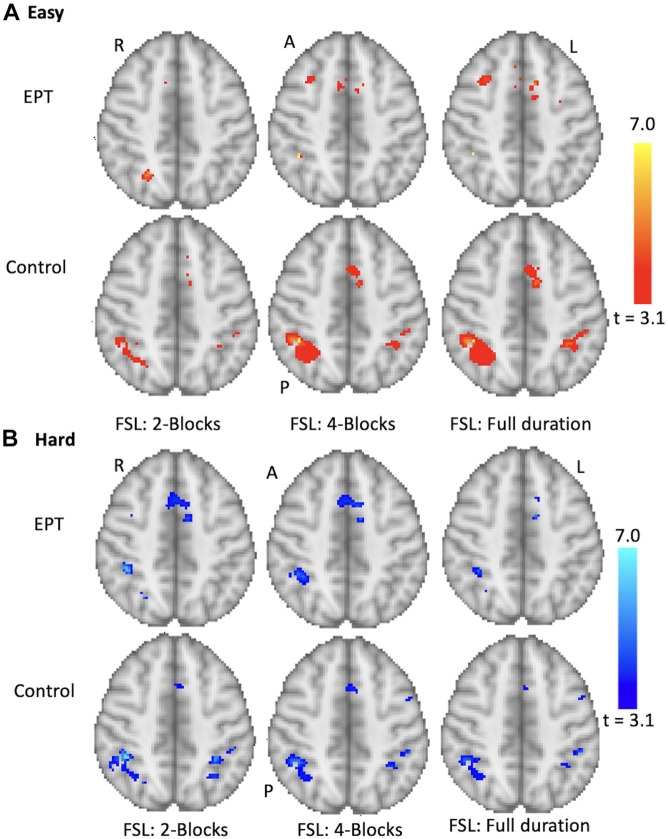
Group results for the 1-back task. Analysis performed for control and EPT subjects using FSL. Early stopping with 2- and 4-blocks being initially administered is compared to full duration. Activations are overlaid on the MNI template brain. Red **(A)** = easy level results, Blue **(B)** hard level results. Group results thresholded at *z* = 3.10 (*p* < 0.001). Slice *z* = 59 is shown. R = right, L = left, A = anterior, P = posterior.

The results for the group 1-back easy and hard contrasts for the 2- and 4-block first stage conditions for EPT and control subjects are shown in Figure 9. Location of activity is listed in Supplementary Table 4. The group results of full scan durations are compared to the group results using only the scans up to the early stopping point for each subject for each difficulty level and number of blocks completed before early stopping was allowed. We examined within-group differences as well as between group differences. The EPT > control (activity that is greater in the EPT group compared to the control group) and control > EPT (activity that is greater in the control group compared to the EPT group) contrasts. The focus for the results here is within-group for the easy and hard levels.

The control subjects show activations in the anterior cingulate and bilateral parietal regions, see Supplementary Table 4, and Figure 9. The cluster sizes and overlap are reported in Table 3. The easy and hard 4-block first stage scans for the control group appear similar to the final scans. There is less correspondence between the 2-block first stage scans and the final scans, reflecting the individual results reported above. The EPT group easy level scans are inconsistent across stages and there is more variability in the activations at both easy and hard levels. This is discussed below.

**TABLE 3:** The number of active voxels that spatially overlap between early stopping and full duration group analyses are listed.

	No of Active Voxels	Voxels in Scan at Early Stopping but not Final Scan	Voxels in Final Scan but not in Scan at Early Stopping	% of Common Voxels with Final Scan	Standard Deviation Values
**EPT – Easy**					
2-Blocks	343	277	284	18.9	137.8
4-Blocks	439	234	145	58.8	92.6
Full duration	350				96.4
**EPT – Hard**					
2-Blocks	1,103	841	339	43.6	113.1
4-Blocks	955	491	137	77.2	98.5
Full duration	601				93.9
**Controls – Easy**					
2-Blocks	1,277	514	1147	39.9	122.3
4-Blocks	1,963	366	313	83.6	84.4
Full duration	1910				89.1
**Controls – Hard**					
2-Blocks	1,976	1111	1065	44.8	103.3
4-Blocks	1,672	326	584	69.7	90.3
Full duration	1930				84.5

*Images thresholded at z = 3.10 (p < 0.001). The percentage of voxels-in-common is given relative to the total number of active voxels detected at full duration. Stopping based on 80% classification at the individual level. Standard deviation values are for the number of active voxels individually.*

## Discussion

Based on analysis of a training sample, we have presented a workflow for the implementation of an adaptive real-time fMRI system that allows for statistically driven dynamic adjustment of experimentation based on voxel-level SPRT. We show that this dynamic and adaptive statistical approach is under some circumstances comparable to corresponding fixed experimental designs in terms of detected activation.

Our implementation illustrates the importance of first analyzing a training sample of full scan data, as we illustrate here. Experts can then review the activation thresholds, first stage duration lengths, stopping rule criteria, and the corresponding activation patterns that arise from early stopping rules, to see if they are plausible neuro-scientifically relative to those observed from longer scans. Certainly, our examples, particularly the group analyses are based on a small-scale neuroimaging study. In general, a larger training sample would provide a stronger basis for making these decisions.

We explored imposing two different first stage lengths before early stopping was considered using either 2- or 4-blocks each of easy and hard stimulus administration. The 4-block first stage is justified over the 2-block because of the comparative stability of the estimation of error variances and other GLM parameters. In contrast, for the 2-block first stage, parameter estimation can be more variable. Also, correspondence in early stop activation patterns to full scan duration involves larger z-score threshold changes over the scans from the initial 3.10-value that was specified, and activation magnitude thresholds for stopping that can be relatively higher. The 2-block first stage often led to most voxels being classified as non-active. See Supplementary Table 2. While the 4-block first stage provides less opportunity for efficiency gains, as the window for early stopping is narrower, but it is more prudent given the need for parameter estimates to stabilize. It is possible that a 3-block initial stage could provide comparable results as the 4-block initial stage, but this was not explored here.

In the SPRT framework, other α_*E*_, *β*_*E*_ pairs were considered as well, to test how different combinations impact activity detection and early stopping. For instance, given selection of α_*E*_ = 0.001 and *β*_*E*_ = 0.01, early stopping was common for the 2-block first stage but arose less often for 4-block first stage and with much smaller savings in scan time. In this case, the more stringent choice of *β*_*E*_ makes it more difficult to cross either of the SPRT thresholds. We also saw that for either α_*E*_ = 0.001 or α_*E*_ = 0.0001 being paired with *β*_*E*_ = 0.1, early stopping occurred for both of the experimental conditions, with somewhat faster early stopping for the less stringent α_*E*_.

In terms of the global stop rule threshold, we observed that for the cases under consideration, stopping when 80% of voxels in the full brain (or smaller ROI), respectively, satisfy their SPRT-based stopping criterion generally leads to early stopping of stimulus administration, while also leading to comparable activation classification as with the full protocol. The stricter 90% criterion was infrequently satisfied, and did not often lead to early stopping of experimentation at 4-blocks first stage estimation. Recall that when GLM parameter values are “in-between” the null and alternative hypothesis values, SPRT-based stopping is less likely at the voxel level. A 100% stopping rule is thus not feasible, as are values relatively close to 100%. The 80% rule seems conservative in that not all participants are stopped early, but there are high levels of correspondence in individual and group level activation maps with full durations, particularly when the first stage is comprised of 4 blocks. The 70% rule is more aggressive, and early stopping is invoked more frequently. Given that the resultant images from early stopping in many cases appear similar across these two rules, the 70% rule should be considered as well.

The SPRT approach was effective at detecting brain activity at the individual level with early stopping in both the control and EPT groups. Note the individual variability among subjects in early stopping performance. Factors that can affect stopping times include the magnitudes of activation, variability in task performance, sustained attention levels, motion, and the noise levels in the BOLD signal. Those born EPT also can have structural abnormalities of the brain which can affect fMRI results and 2 subjects reported here had clear abnormalities that were obvious even in this low-resolution fMRI data (1.8 mm × 1.8 mm × 4.0 mm voxel size). Subtle abnormalities may have been present in some of the other subjects.

The EPT group data demonstrated less consistent activity by comparison to control group data across all stopping points. In order to understand this result it is necessary to consider neuropsychological skills and structural and functional brain changes within the group. Working memory is a key skill required for both mathematics and this numerical 1-back task. Recall the lower accuracy and longer response times in the EPT group. fMRI studies on dyscalculia (difficulty in learning and performing mathematics) suggest that there is greater heterogeneity in activations with a more diffuse pattern being apparent (Berl et al., 2006; Kucian et al., 2006). Additionally, there is overlap in structural differences in white matter integrity, as measured from diffusion weighted imaging studies, between those born EPT and those with dyscalculia including inferior fronto-occipital fasciculus and the inferior and superior longitudinal fasciculi (Rykhlevskaia et al., 2009; Kucian et al., 2014; Young et al., 2017; Loe et al., 2019). These connect crucial areas associated with mathematics and working memory. A more diffuse and variable pattern of functional activity, perhaps partly due to structural differences, may confound a group analysis in this instance. The control group variances are relatively much lower throughout, as the extremely premature birth group was neurologically and cognitively more heterogeneous. If group-level analysis is a main objective, it is possible that groups could be treated differently in how early stopping is approached based on within-group heterogeneity and the need for more scan data to help overcome this. This issue needs further investigation. Certainly, relatively larger group sizes for patient groups also would be helpful in overcoming greater within-group variability.

Based on our analysis of head movements, early stopping of data collection could be particularly useful in populations where attention is problematic, such as with young children or adults with cognitive deficits. We see in our EPT population that head movement became more frequent and had a greater amplitude in the second half of the scans when compared to the control subjects. This may be an indication of attentional fatigue. In Table 3, it is seen for the easy level, the number of voxels classified as active actually decreases a bit at full scan duration compared with early stopping. This may be due to relative increases in variability from loss of attention. In general, we believe that there will be instances when early stopping can actually improve statistical power by reducing the possibility of fatigue.

In the future, it is possible that the first stage length can be tailored at the voxel level, once it is clear error variance and other GLM parameter estimates are relatively stable, which is expected at some point due to the convergence properties of the estimators. This may facilitate earlier stopping. Alternatively, if local computational resources are limited, note that stopping can be assessed on an interval basis, and not necessarily after every scan. Although not considered here, these BOLD signal-based early stopping rules could also possibly be enriched by incorporating individual motion displacement patterns, as well as behavioral measures such as correctness rates in experimentation.

Here we demonstrated full brain analytics with parallelization using MKL Intel libraries for matrix computation with two Xeon E5-2687W 8-core processors. It is also feasible to consider only partial brain volumes where experiments demand more consideration of a particular area. Future directions for the study are to implement the SPRT and Bayesian sequential estimation methods using distributed computing approaches to increase processing speed and allow advance in stopping rule methods under shorter scan TR periods.

## Conclusion

We introduce a systematic, statistically based approach to dynamic experimentation with real-time fMRI. Saving in scan time and accurate voxel activation detection can be achieved, while redundant experimentation in block design is reduced. We investigate different aspects of how to determine early stopping rules. These analyses can be viewed as intended on a training sample to guide implementation of early stopping in future studies involving the same experiments and study populations. These methods can be particularly useful such as when investigating fragile patients and young children, as shorter scan requirements can enhance patient experience and reduce fatigue effects. Another possible application is in real-time quality control, where it is important to know if sufficient testing has been conducted, or whether more experimentation is needed. These methods lay a foundation for future dynamic experimentation approaches and with real-time fMRI, including for resting state, neurofeedback and presurgical evaluation. Use of high-performance computing will enable the advent of more sophisticated real-time experimental designs and dynamically determined early stopping rules.

## Data Availability Statement

The raw data supporting the conclusions of this article will be made available by the authors, without undue reservation.

## Ethics Statement

The studies involving human participants were reviewed and approved by University Hospitals Cleveland Medical Center Institutional Review Board. Written informed consent to participate in this study was provided by the participants’ legal guardian/next of kin.

## Author Contributions

SC and CT contributed to study design, analysis and interpretation of data, and drafting of manuscript. WC contributed to study design, analysis and interpretation of data, software development, and drafting of manuscript. JF contributed to study design and software development. HF contributed to study design and technical support, and drafting of manuscript. JS-G contributed to software development and revision of manuscript. JZ contributed to analysis and interpretation of data, and revision of manuscript. All authors contributed to the article and approved the submitted version.

## Conflict of Interest

HF was employed by the company Philips Healthcare, USA. JS-G was employed by the company Philips Healthcare, Spain. The remaining authors declare that the research was conducted in the absence of any commercial or financial relationships that could be construed as a potential conflict of interest. The authors declare that this study received funding from Philips Healthcare. The funder had the following involvement in the study: technical support and software development for the real-time output of fMRI files and revision of the manuscript.

## Publisher’s Note

All claims expressed in this article are solely those of the authors and do not necessarily represent those of their affiliated organizations, or those of the publisher, the editors and the reviewers. Any product that may be evaluated in this article, or claim that may be made by its manufacturer, is not guaranteed or endorsed by the publisher.

## Footnotes

1
www.proedinc.com

2
https://www.python.org/

3
https://bitbucket.org/tatsuoka-lab/fmri-presentation

4
https://afni.nimh.nih.gov

5
https://fsl.fmrib.ox.ac.uk/fsl/fslwiki/
