# Supplementary material for: Early Stopping in Experimentation With Real-Time Functional Magnetic Resonance Imaging Using a Modified Sequential Probability Ratio Test
Source: Front Neurosci. 2021 Nov 4;15:643740. doi: 10.3389/fnins.2021.643740 (PMC8600259; doi:10.3389/fnins.2021.643740)
Supplement: Supplementary file 1 [file Table_1.docx]

**Table S1:** Statistical maps of activation for easy and hard levels for the one back task. Maps shown are for those instances where early stopping occurred and there is < 50% overlap between the active voxels of the early stopped scans and the full duration scan. 70% and 80% of voxels classified active and non-active are shown side by side for comparison. Full duration active voxels are shown in blue for all time points for easy reference. 2-block first stage result is overlaid in red, 4-block first stage result is in green. Slice number is given in MNI space. Grey matter is highlighted in yellow.

| **Sub** | **Level** | **SliceZ** | **2-blocks (red)**  **70%** | **2-blocks (red)**  **80%** | **Full duration** | **4-blocks (green)**  **70%** | **4-blocks (green)**  **80%** | **Full duration (blue)** |
| --- | --- | --- | --- | --- | --- | --- | --- | --- |
| **Control** | |  |  |  |  |  |  |  |
| **1** | Easy | 57 | Scan 79 | Scan 121 | Scan 238 |  |  |  |
|  |  |  | 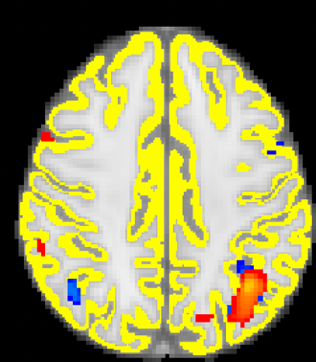 | 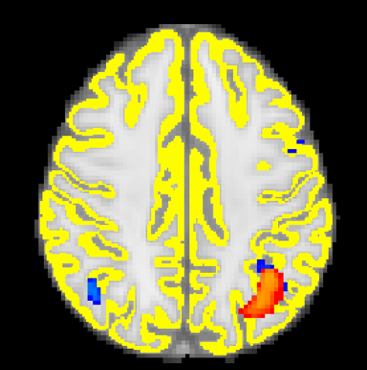 | 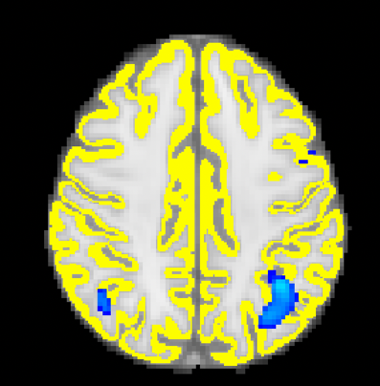 |  |  |  |
|  | Hard | 57 | Scan 79 | Scan 80 | Scan 238 |  |  |  |
|  |  |  | 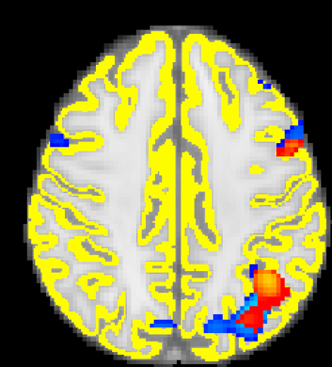 | 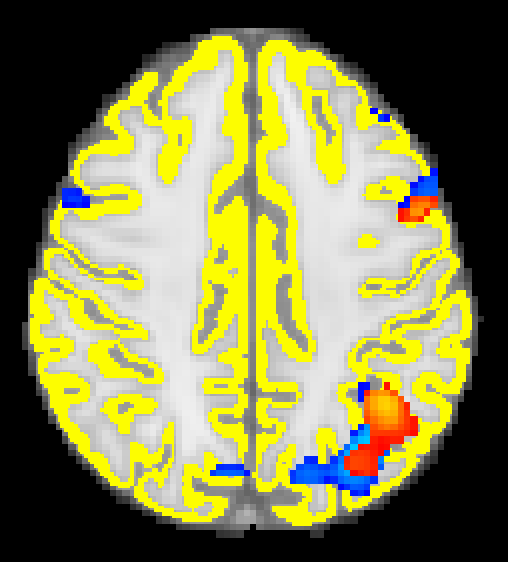 | 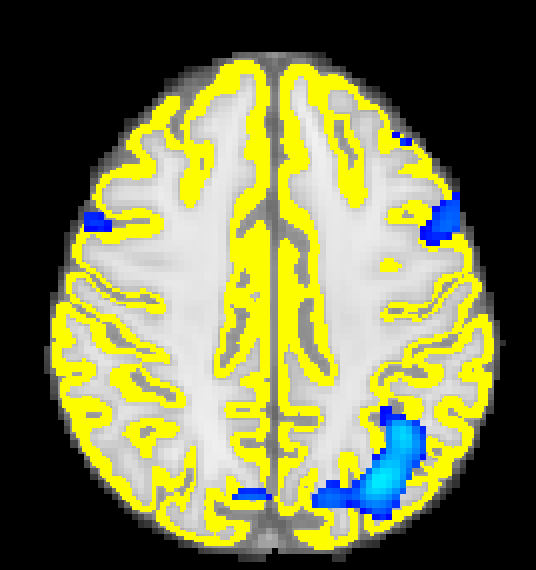 |  |  |  |
|  |  |  |  |  |  |  |  |  |
| **2** | Easy | 57 | Scan 79 | Scan 83 | Scan 238 |  |  |  |
|  |  |  | 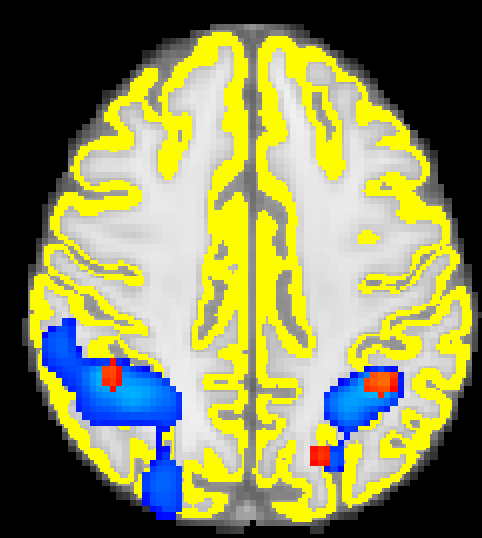 | 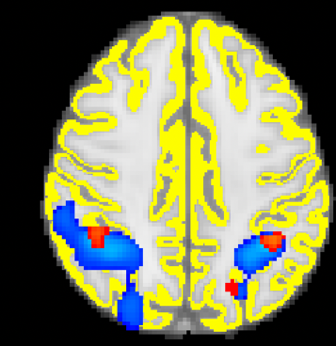 | 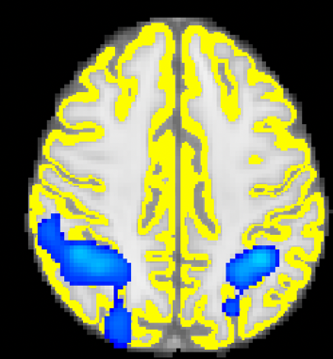 |  |  |  |
|  |  |  |  |  |  |  |  |  |
| **3** | Easy | 52 | Scan 79 | Scan 89 | Scan 238 |  |  |  |
|  |  |  | 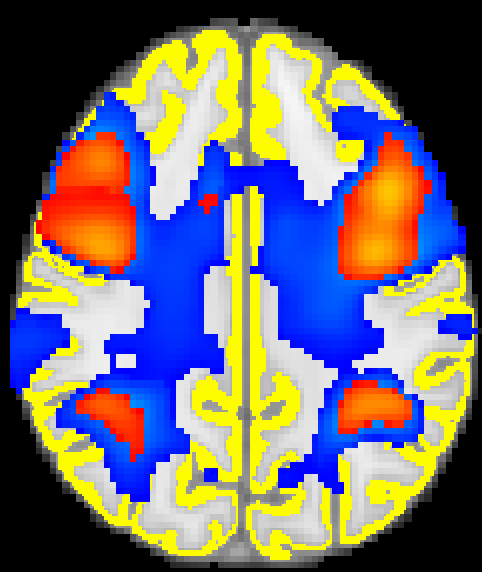 | 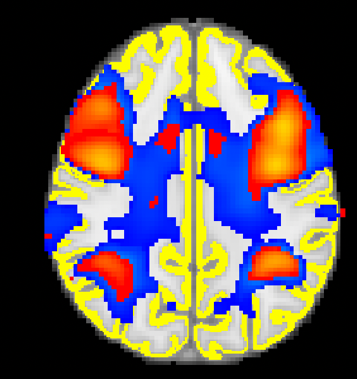 | 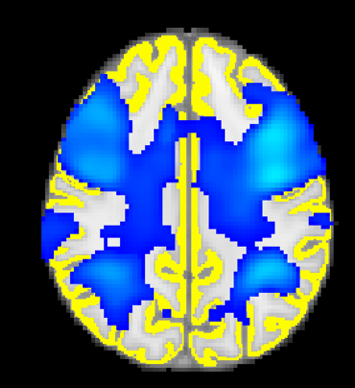 |  |  |  |
|  | Hard | 52 | Scan 79 | Scan 79 | Scan 238 |  |  |  |
|  |  |  | Same as 80% | 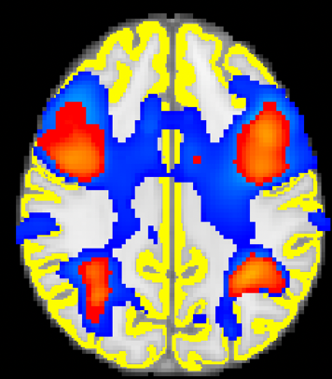 | 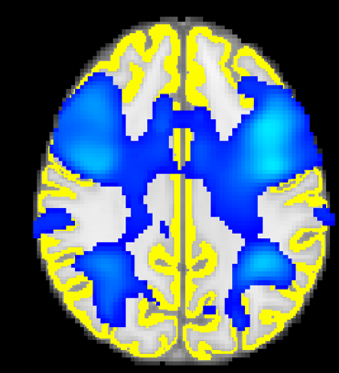 |  |  |  |
|  |  |  |  |  |  |  |  |  |
| **4** | Hard | 52 | Scan 79 | Scan 84 | Scan 238 |  |  |  |
|  |  |  | 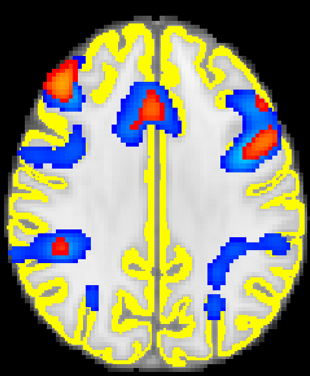 | 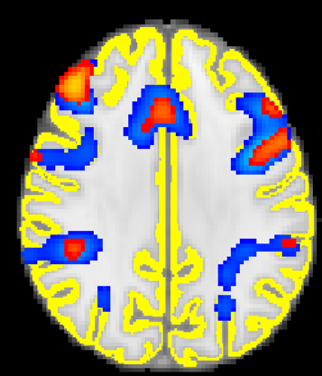 | 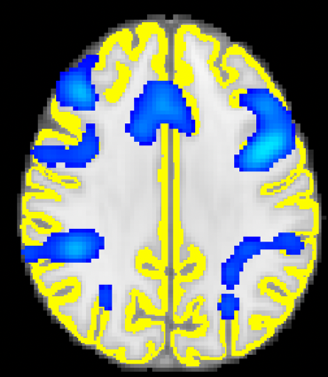 |  |  |  |
|  |  |  |  |  |  |  |  |  |
| **5** | Easy | 57 | Scan 79 | Scan 100 | Scan 238 |  |  |  |
|  |  |  | 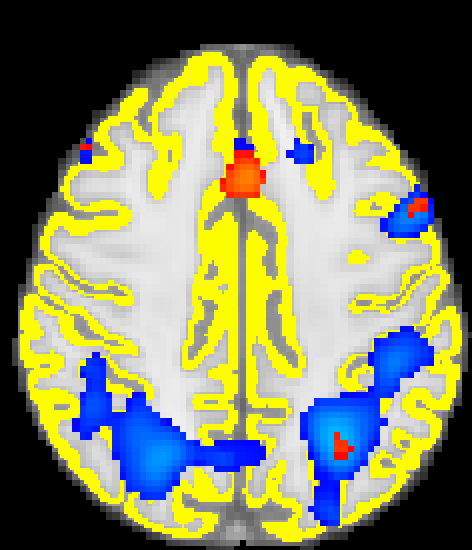 | 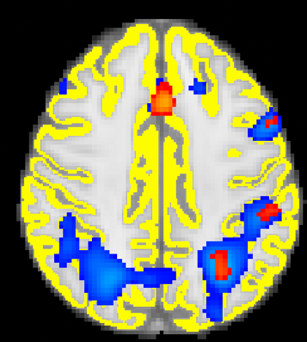 | 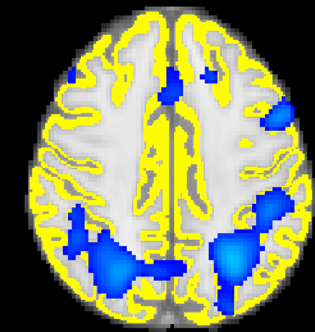 |  |  |  |
|  | Hard | 55 | Scan 79 | Scan 79 | Scan 238 |  |  |  |
|  |  |  | Same as 80% | 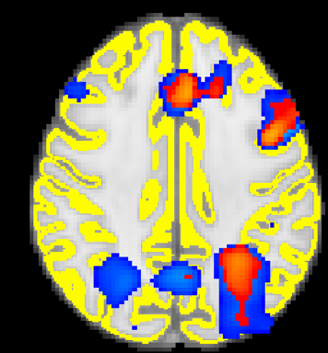 | 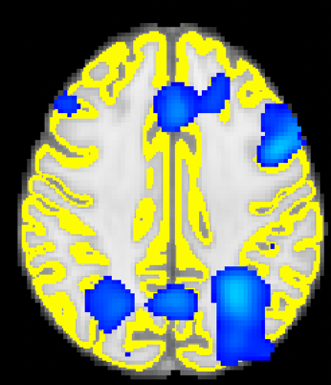 |  |  |  |
|  |  |  |  |  |  |  |  |  |
| **6** | Easy | 54 | Scan 79 | Scan 79 | Scan 238 |  |  |  |
|  |  |  | Same as 80% | 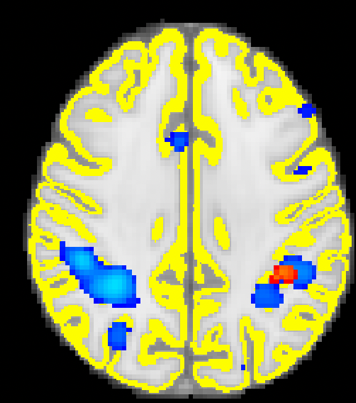 | 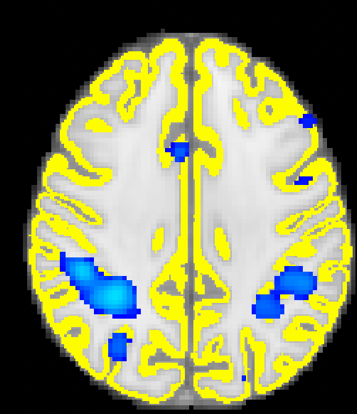 |  |  |  |
|  | Hard | 54 | Scan 85 | Scan 87 | Scan 238 |  |  |  |
|  |  |  | 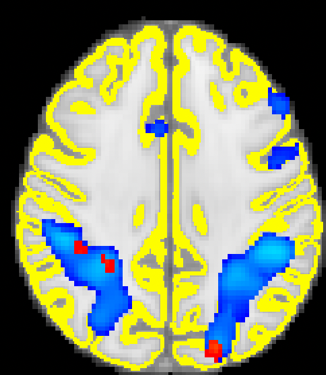 | 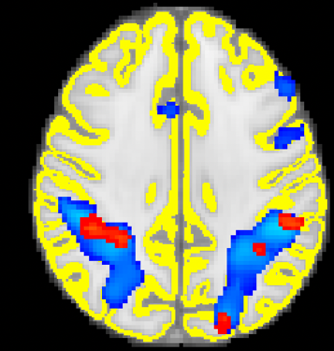 | 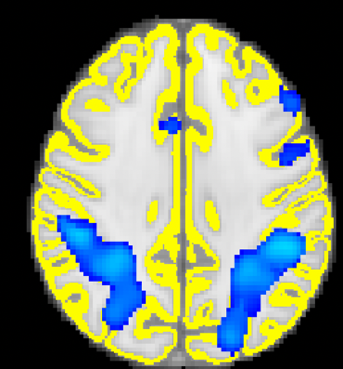 |  |  |  |
|  |  |  |  |  |  |  |  |  |
| **7** | Easy | 54 | Scan 79 | Scan 79 | Scan 238 | Scan 155 | Scan 164 | Scan 238 |
|  |  |  | Same as 80% | Virtually no overlap to  show visually | 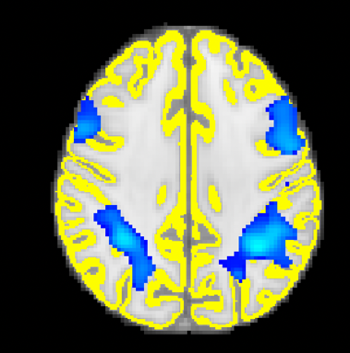 | 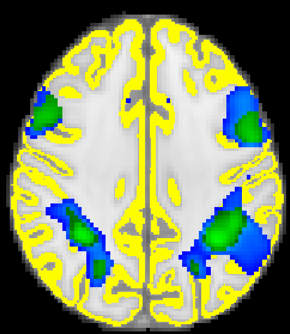 | 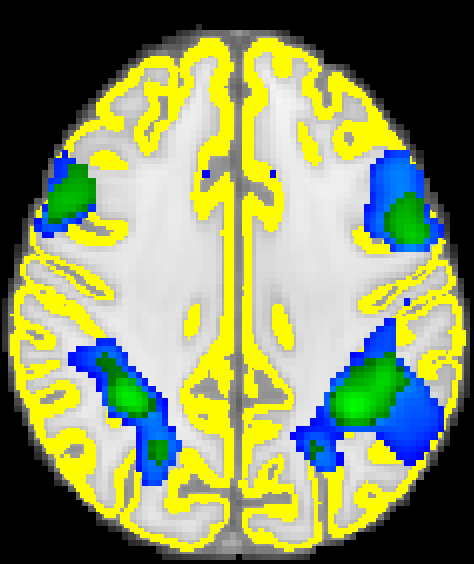 | 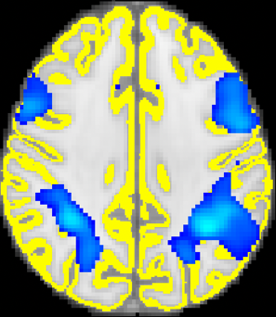 |
|  | Hard | 54 | Scan 80 | Scan 85 | Scan 238 | Scan 155 | Scan 155 | Scan 238 |
|  |  |  | 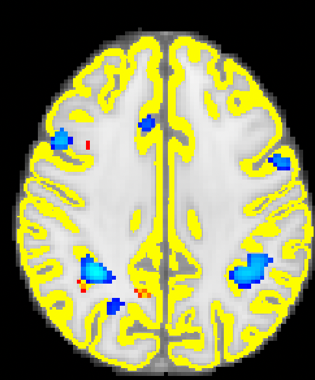 | 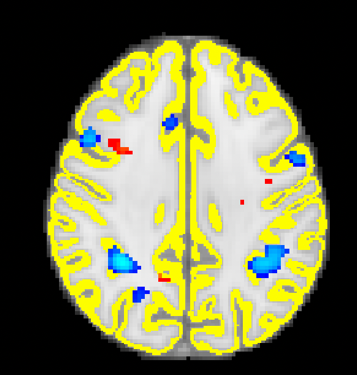 | 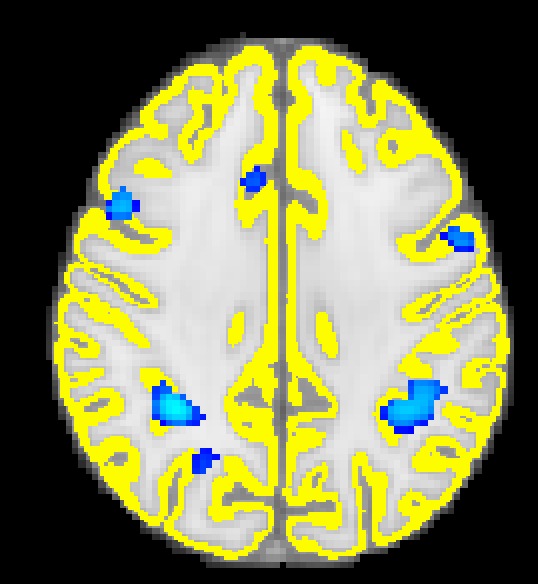 | Same as 80% | 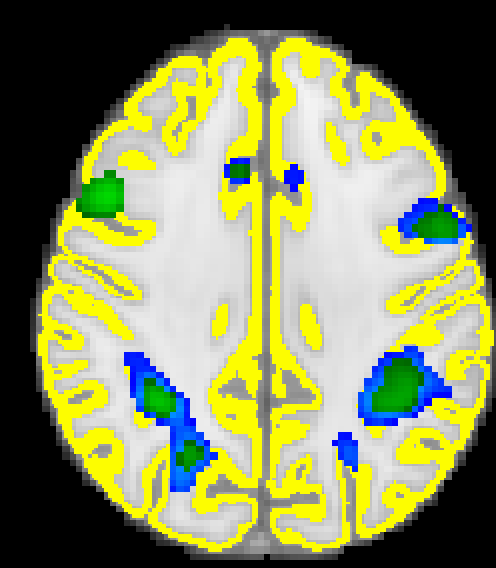 | 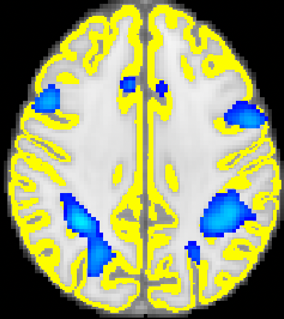 |
|  |  |  |  |  |  |  |  |  |
| **8** | Hard | 50 | Scan 79 | Scan 97 | Scan 238 |  |  |  |
|  |  |  | 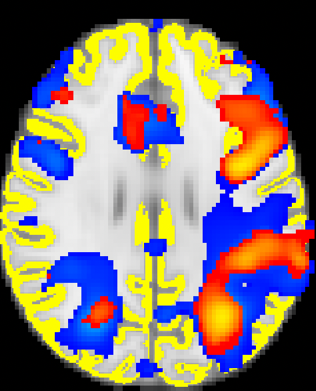 | 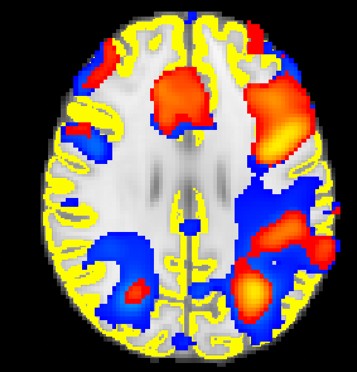 | 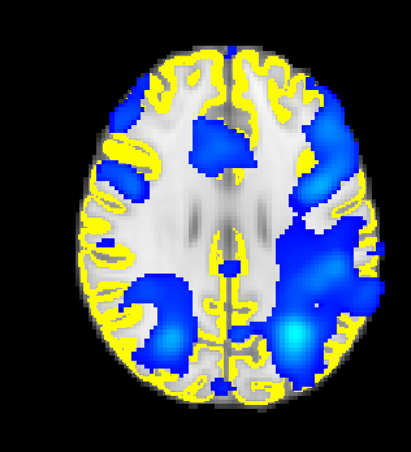 |  |  |  |
|  |  |  |  |  |  |  |  |  |
| **9** | Easy | 54 | Scan 79  Same as 80% | Scan 79  Virtually no overlap to  show visually | Scan 238  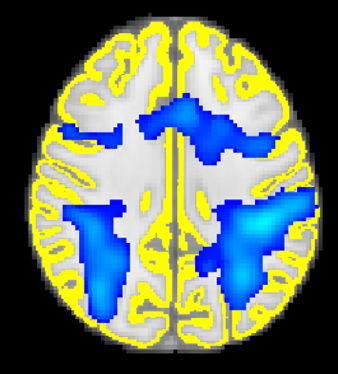 | Scan 155  Same as 80% | Scan 155  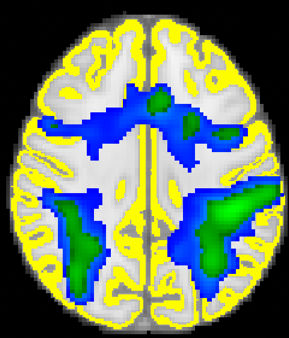 | Scan 238  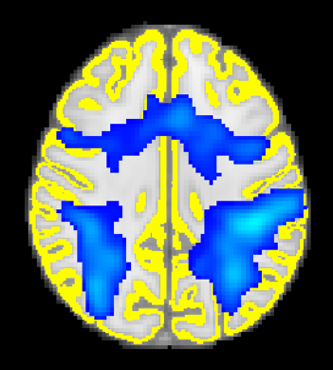 |
|  | Hard | 57 |  |  |  | Scan 155  Same as 80% | Scan 155  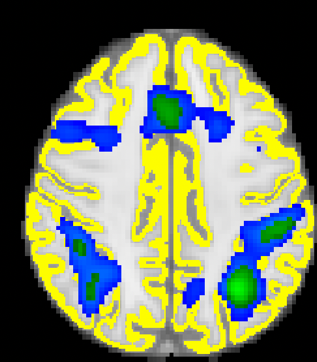 | Scan 238  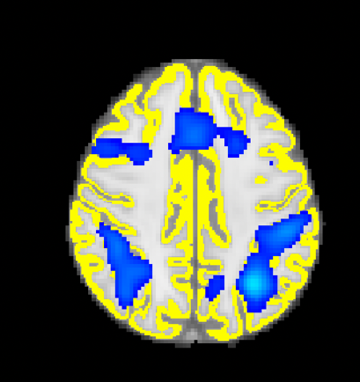 |
|  |  |  |  |  |  |  |  |  |
| **10** | Easy | 50 | Scan 79  Virtually no overlap to show visually | Scan 103  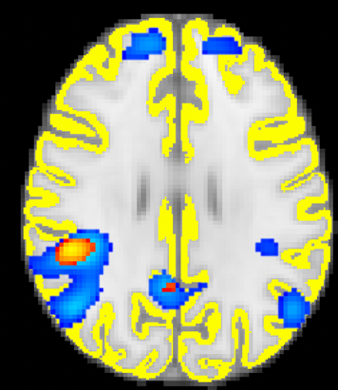 | Scan 238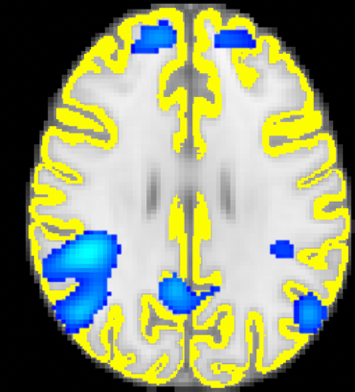 |  |  |  |
|  | Hard | 51 | Scan 79 | Scan 80 | Scan 238 | Scan 155 | Scan 155 | Scan 238 |
|  |  |  | 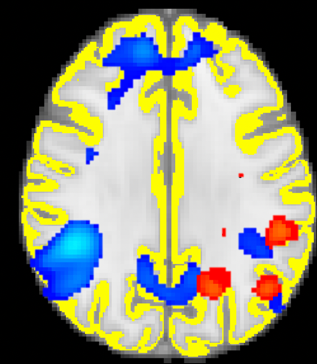 | 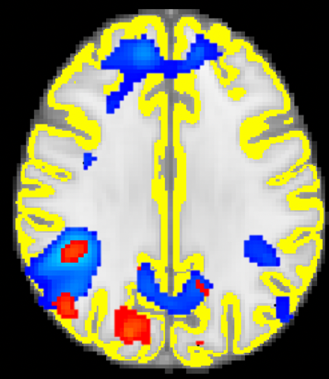 | 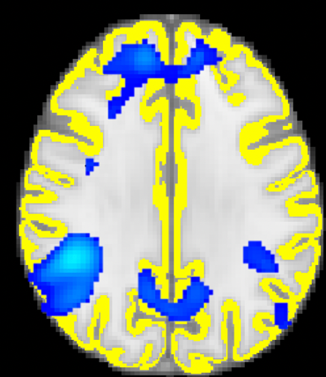 | Same as 80% | 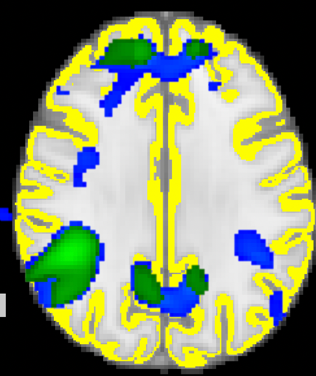 | 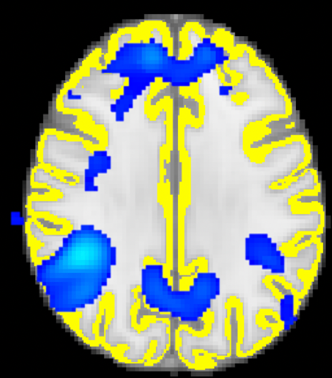 |
|  |  |  |  |  |  |  |  |  |
| **11** | Easy | 53 | Scan 79  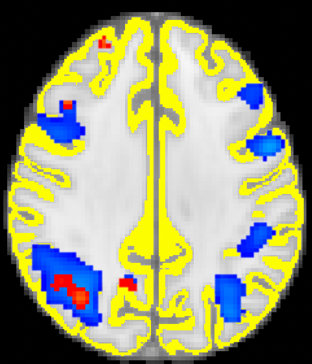 | Scan 86  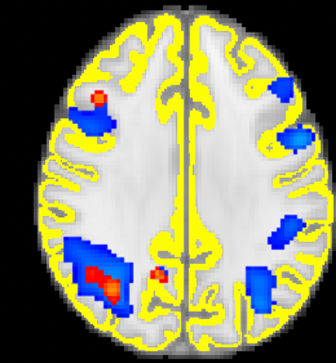 | Scan 238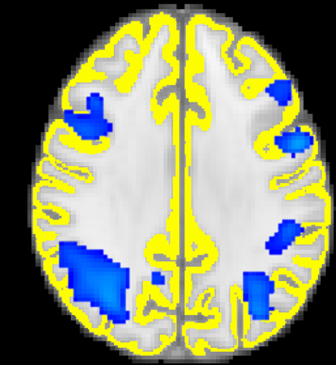 | Scan 155  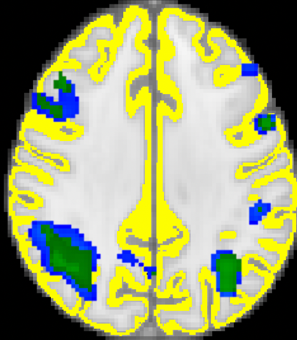 | Scan 166  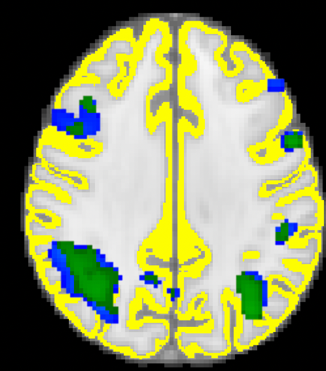 | Scan 238  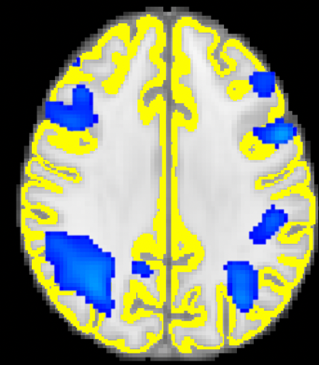 |
|  | Hard | 52 | Scan 80 | Scan 110 | Scan 238 |  |  |  |
|  |  |  | 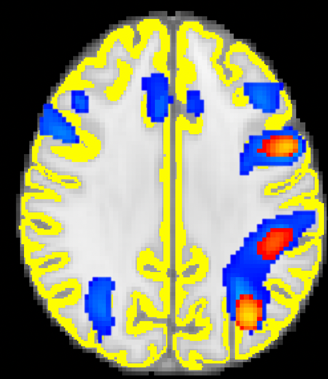 | 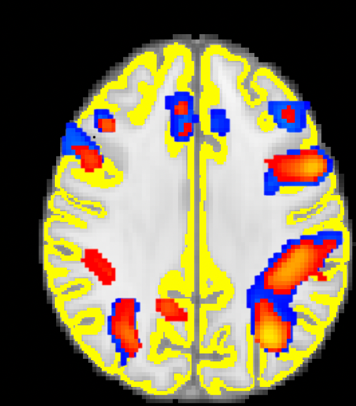 | 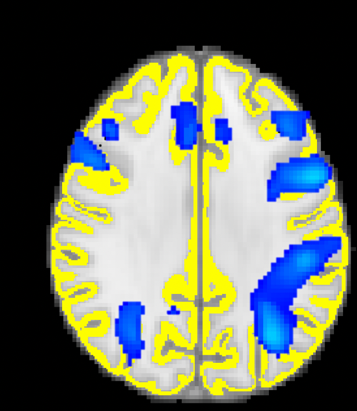 |  |  |  |
|  |  |  |  |  |  |  |  |  |
| **12** | Easy | 52 | Scan 79 | Scan 104 | Scan 238 |  |  |  |
|  |  |  | 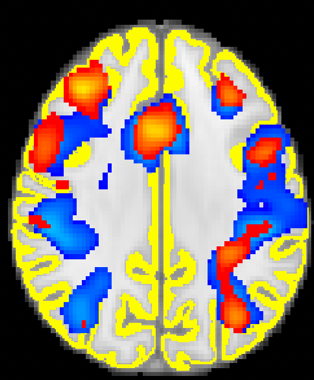 | 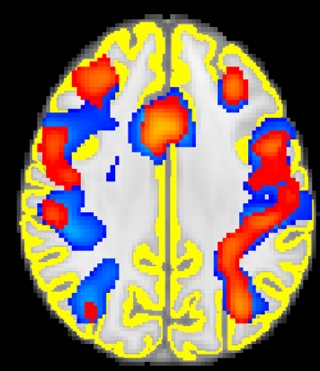 | 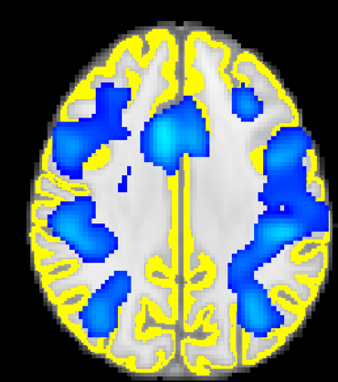 |  |  |  |
|  | Hard | 55 | Scan 79 | Scan 98 | Scan 238 |  |  |  |
|  |  |  | 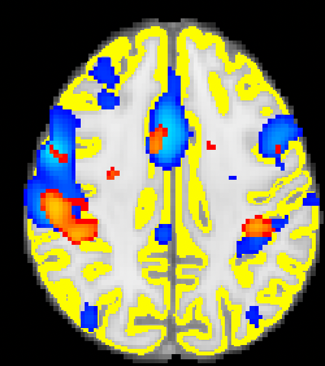 | 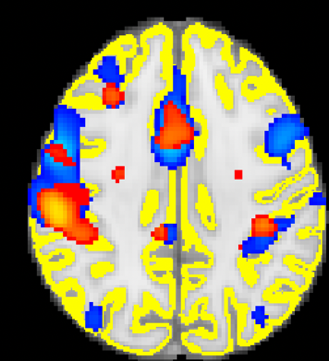 | 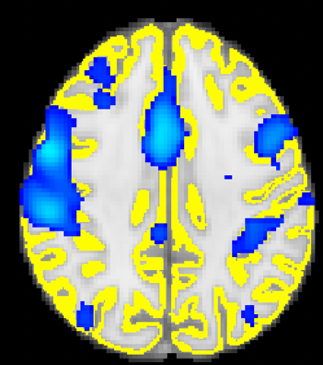 |  |  |  |
|  |  |  |  |  |  |  |  |  |
| **EPT** |  |  |  |  |  |  |  |  |
| **13** | Easy |  | Scan 79 | Scan 79 | Scan 238 | Scan 155 | Scan 155 | Scan 238 |
|  |  |  | Same as 80% | Virtually no overlap to  show visually | Virtually no activity to show visually | Same as 80% | Virtually no overlap to  show visually | Virtually no activity to show visually |
|  | Hard | 57 | Scan 79 | Scan 79 | Scan 238 | Scan 155 | Scan 155 | Scan 238 |
|  |  |  | Same as 80% | Virtually no overlap to  show visually | Virtually no activity to show visually | Same as 80% | 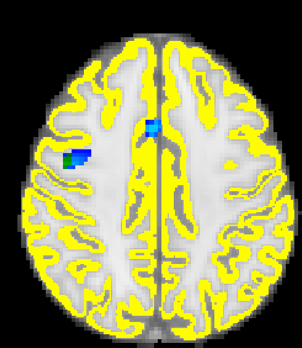 | 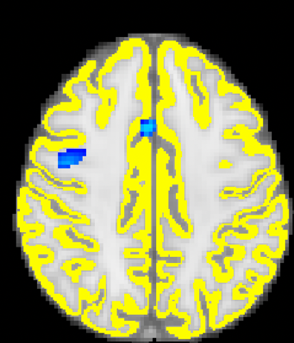 |
|  |  |  |  |  |  |  |  |  |
| **14** | Easy | 56 | Scan 79 | Scan 104 | Scan 238 |  |  |  |
|  |  |  | 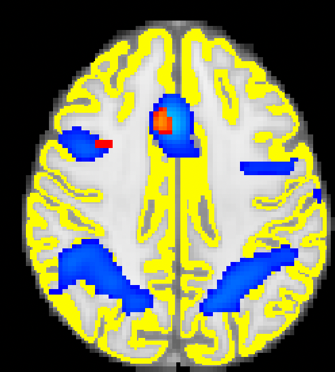 | 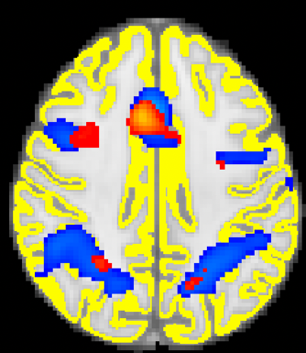 | 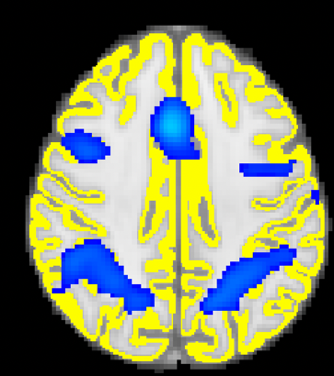 |  |  |  |
|  | Hard |  | Scan 79 | Scan 79 | Scan 238 |  |  |  |
|  |  |  | Same as 80% | 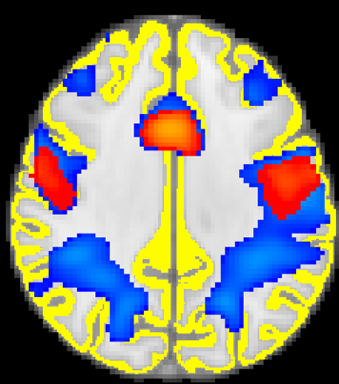 | 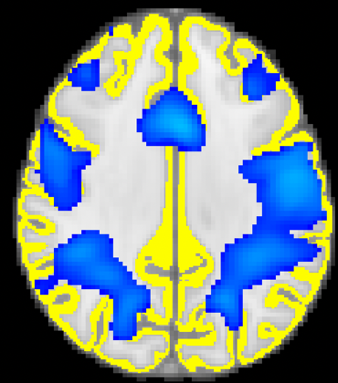 |  |  |  |
|  |  |  |  |  |  |  |  |  |
| **15** | Easy | 54 | Scan 103 | Scan 105 | Scan 238 |  |  |  |
|  |  |  | Virtually no overlap to show visually | Virtually no overlap to  show visually | Virtually no activity to show visually |  |  |  |
|  | Hard | 48 | Scan 79 | Scan 84 | Scan 238 |  |  |  |
|  |  |  | 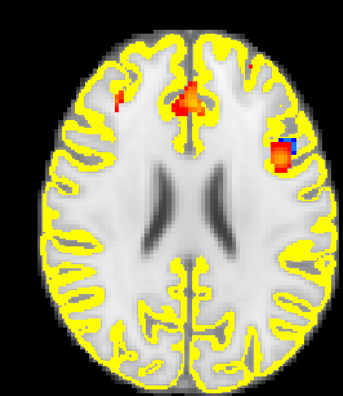 | 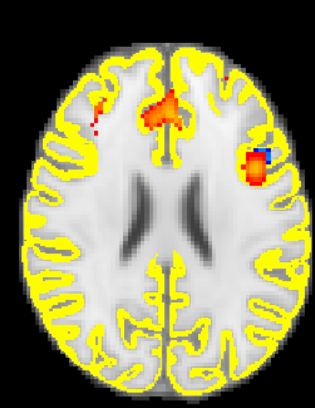 | 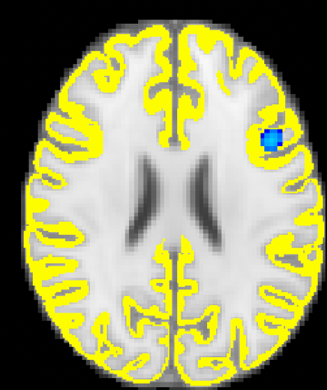 |  |  |  |
|  |  |  |  |  |  |  |  |  |
| **16** | Hard | 59 | Scan 85 | Scan 99 | Scan 238 |  |  |  |
|  |  |  | 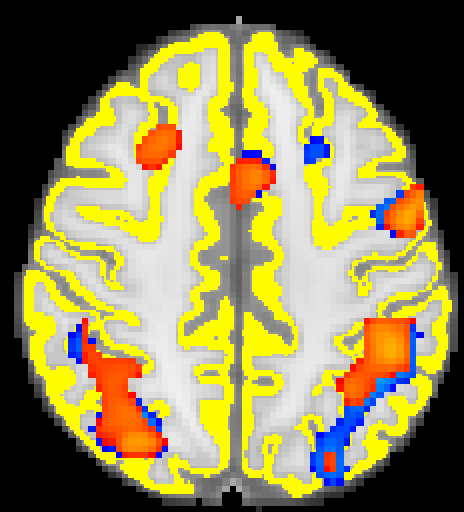 | 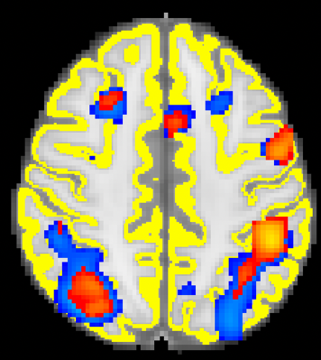 | 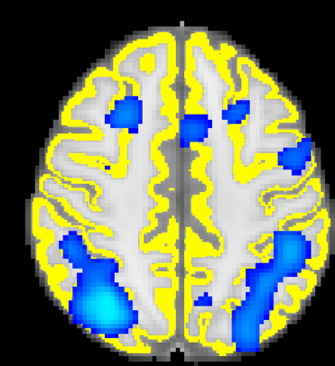 |  |  |  |
|  |  |  |  |  |  |  |  |  |
| **17** | Easy | 58 | Scan 79  Same as 80% | Scan 79  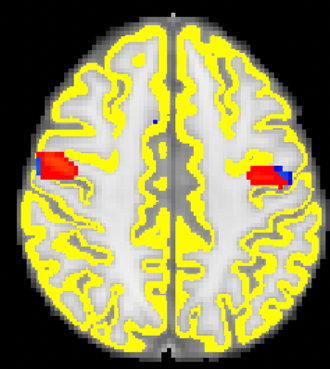 | Scan 238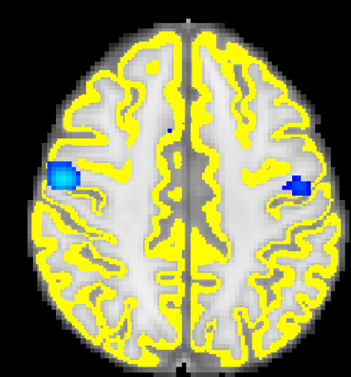 |  |  |  |
|  | Hard | 51 | Scan 79  Same as 80% | Scan 79  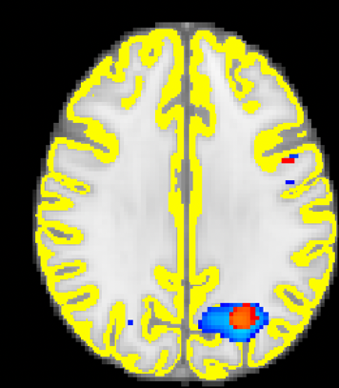 | Scan 238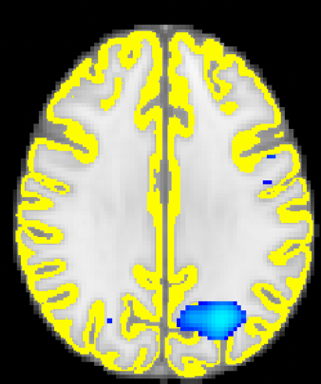 |  |  |  |
|  |  |  |  |  |  |  |  |  |
| **18** | Easy | 53 | Scan 79  Same as 80% | Scan 79  Virtually no overlap to  show visually | Scan 238  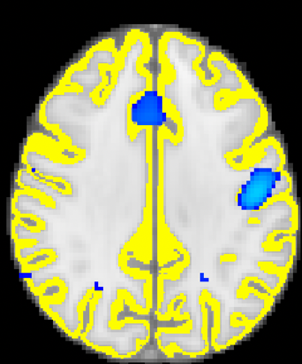 | Scan 155  Same as 80% | Scan 155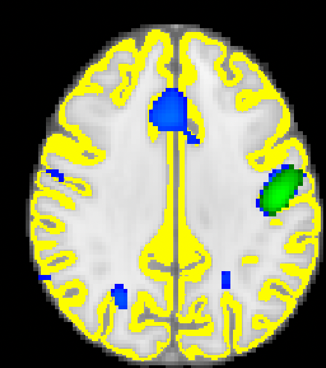 | Scan 238  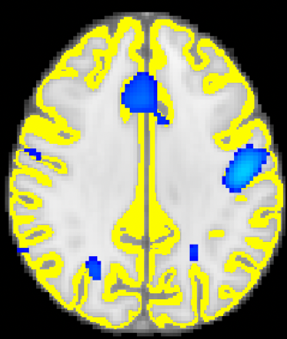 |
|  |  |  |  |  |  |  |  |  |
|  | Hard | 55 | Scan 87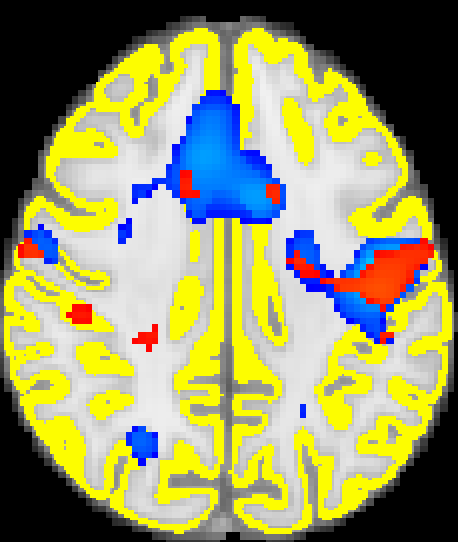 | Scan 119  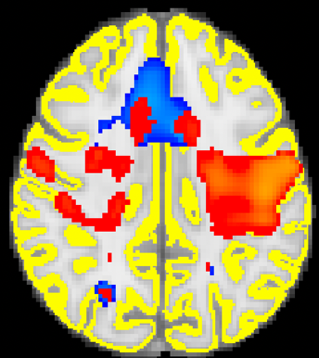 | Scan 238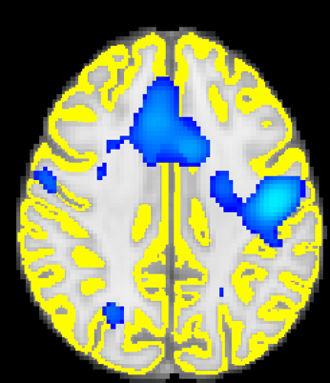 |  |  |  |
|  |  |  |  |  |  |  |  |  |
| **19** | Easy | 57 | Scan 79  Virtually no overlap to show visually | Scan 85  Virtually no overlap to  show visually | Scan 238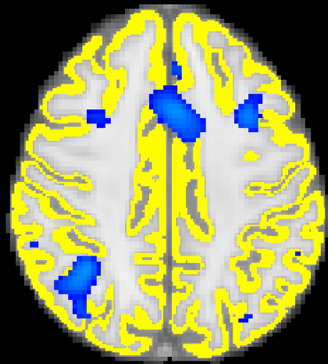 |  |  |  |
|  | Hard | 63 | Scan 79  Same as 80% | Scan 79  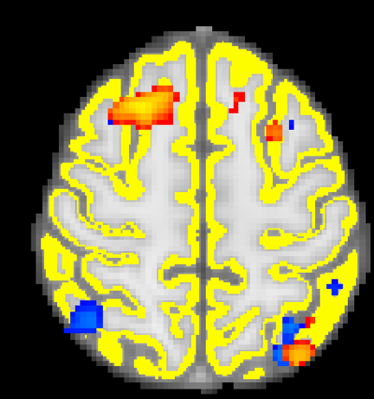 | Scan 238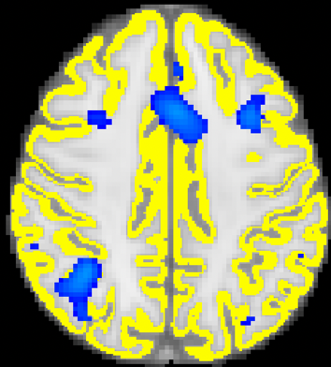 | Scan 155  Same as 80% | Scan 155  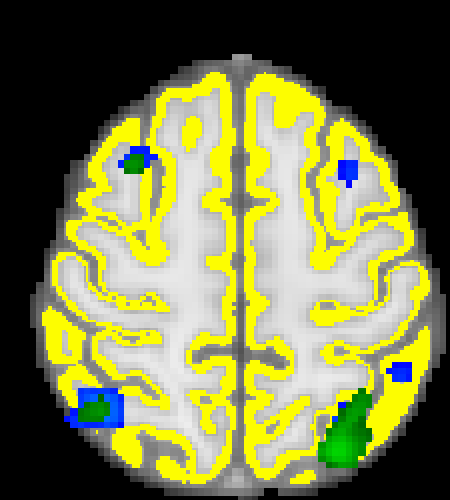 | Scan 238  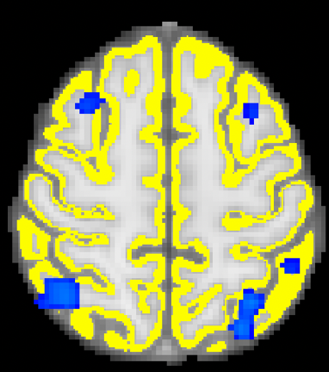 |
|  |  |  |  |  |  |  |  |  |
| **20** | Easy | 54 | Scan 79  Same as 80% | Scan 79  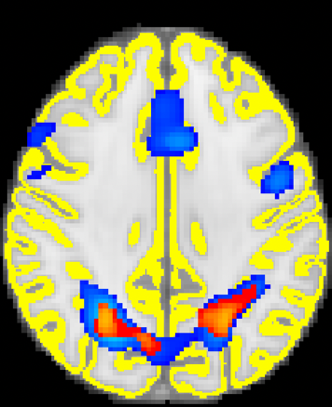 | Scan 238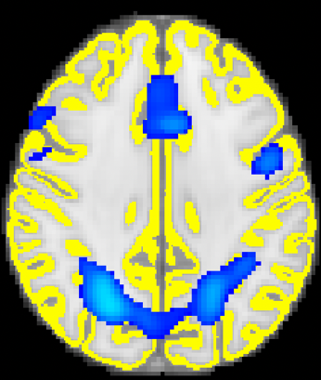 |  |  |  |
|  | Hard | 54 | Scan 79  Same as 80% | Scan 79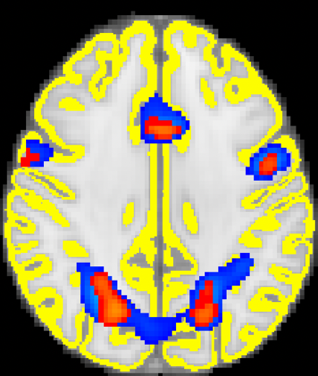 | Scan 238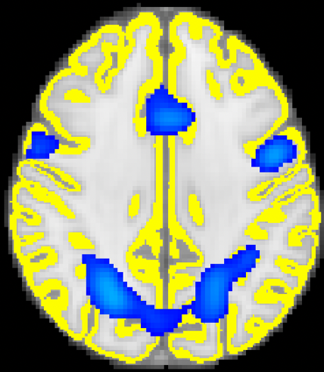 |  |  |  |
| **21** | Hard | 59 | Scan 79 | Scan 80 | San 238 |  |  |  |
|  |  |  | 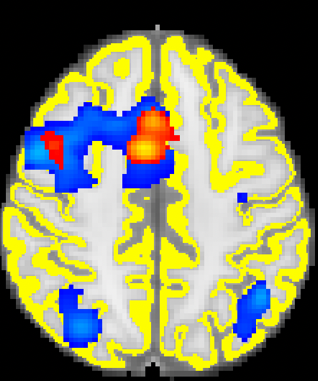 | 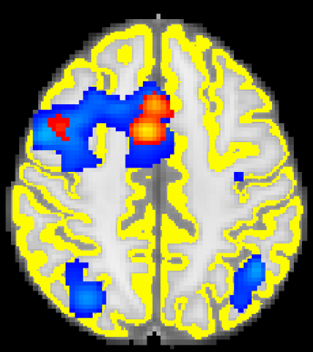 |  |  |  |  |
|  |  |  |  |  |  |  |  |  |
| **22** | Easy | 53 | Scan 89 | Scan 107 | Scan 238 |  |  |  |
|  |  |  | Virtually no overlap to show visually |  |  |  |  |  |
|  | Hard | 54 | Scan 79 | Scan 84 | Scan 238 |  |  |  |
|  |  |  |  |  |  |  |  |  |

**Table S2:** Plots of the percentage of voxels classified as active and non-active at each scan number for each difficulty level for each subject. Information is given from scan 79 to 238.

| **Subject** | **Total number of voxels** | **First stage estimation** | **Classification of voxels by scan number** |
| --- | --- | --- | --- |
| **Control** |  |  |  |
| **1** | 115,062 | 2-Block |  |
|  |  | 4-Block |  |
| **2** | 113,564 | 2-block |  |
|  |  | 4-Block |  |
| **3** | 77,359 | 2-block |  |
|  |  | 4-Block |  |
| **4** | 103,591 | 2-block |  |
|  |  | 4-Block |  |
| **5** | 114,260 | 2-block |  |
|  |  | 4-Block |  |
| **6** | 121,353 | 2-block |  |
|  |  | 4-Block |  |
| **7** | 107,406 | 2-block |  |
|  |  | 4-Block |  |
| **8** | 106,267 | 2-block |  |
|  |  | 4-Block |  |
| **9** | 121,195 | 2-block |  |
|  |  | 4-Block |  |
| **10** | 96,565 | 2-block |  |
|  |  | 4-Block |  |
| **11** | 107,016 | 2-block |  |
|  |  | 4-Block |  |
| **12** | 96,936 | 2-block |  |
|  |  | 4-Block |  |
|  |  |  |  |
| **EPT** |  |  |  |
| **13** | 94,623 | 2-block |  |
|  |  | 4-Block |  |
| **14** | 94,905 | 2-block |  |
|  |  | 4-Block |  |
| **15** | 98,799 | 2-block |  |
|  |  | 4-Block |  |
| **16** | 118,098 | 2-block |  |
|  |  | 4-Block |  |
| **17** | 124,749 | 2-block |  |
|  |  | 4-Block |  |
| **18** | 97,437 | 2-block |  |
|  |  | 4-Block |  |
| **19** | 135,379 | 2-block |  |
|  |  | 4-Block |  |
| **20** | 89,609 | 2-block |  |
|  |  | 4-Block |  |
| **21** | 104,584 | 2-block |  |
|  |  | 4-Block |  |
| **22** | 114,201 | 2-block |  |
|  |  | 4-Block |  |
| **23** | 86,177 | 2-block |  |
|  |  | 4-Block |  |

**Table S3:** Plots of framewise displacement and subject responses. 1^st^ panel (top): framewise displacement estimation from motion parameters. 2^nd^ panel: Subject responses for easy level, y-axis magnitude indicates time taken to respond. 3^rd^ Panel: hard level subject responses, y-axis magnitude indicates time taken to respond. 4^th^ panel (bottom): the duration of the easy and hard level blocks are shown for reference along with the timing of the expected responses during each block (‘Exp’ – timing of the expected response).

| **Subject** | **Motion and response plots** |
| --- | --- |
| **Control** |  |
| **1** |  |
| **2** |  |
| **3** |  |
| **4** |  |
| **5** |  |
| **6** |  |
| **7** |  |
| **8** |  |
| **9** |  |
| **10** |  |
| **11** |  |
| **12** |  |
|  |  |
| **EPT** |  |
| **13** |  |
| **14** |  |
| **15** |  |
| **16** |  |
| **17** |  |
| **18** |  |
| **19** |  |
| **20** |  |
| **21** |  |
| **22** |  |
| **23** |  |

**Table S4:** List of activations from a group analysis using FSL. Easy and hard levels for control and EPT subjects are reported for early stopping and full scan durations. Thresholded at z = 3.10 and a minimum cluster extent = 15 voxels has been applied.

| **Group** | **Cluster No.** | **Coordinates (MNI)** | **No. of Voxels** | **Peak z-score** | **Side** | **Location** |
| --- | --- | --- | --- | --- | --- | --- |
| **2-Block**  **Early stopping:** | |  |  |  |  |  |
| **Easy** |  |  |  |  |  |  |
| **EPT** | 1  2  3  4  5  6  7 | -6 -2 66  -34 28 26  -40 8 0  24 -58 46  -6 -2 52  -56 10 4  8 4 56 | 58  49  48  46  19  15  15 | 4.47  5.01  4.66  5.24  3.93  5.15  4.23 | Left  Left  Left  Right  Left  Left  Right | Medial Frontal Gyrus  Middle Frontal Gyrus  Insula  Precuneus  Medial Frontal Gyrus  Precentral Gyrus  Medial Frontal Gyrus |
|  |  |  |  |  |  |  |
| **Control** | 1  2  3  4  5  6  7  8  9  10 | -38 2 28  -12 16 32  32 -62 48  -42 -40 36  14 20 32  -6 10 52  16 -36 14  -34 28 26  -28 38 26  30 -46 38 | 415  155  142  123  64  57  55  25  21  15 | 5.76  7.39  5.54  4.83  4.71  5.1  3.97  4.83  4.32  4.2 | Left  Left  Right  Left  Right  Left  Right  Left  Left  Right | Precentral Gyrus  Cingulate Gyrus  Superior Parietal Lobule  Supramarginal Gyrus  Cingulate Gyrus  Medial Frontal Gyrus  Thalamus  Middle Frontal Gyrus  Middle Frontal Gyrus  Cingulate Gyrus |
| **Hard** |  |  |  |  |  |  |
| **EPT** | 1  2  3  4  5  6  7  8  9  10  11 | -4 -2 52  -6 2 64  26 -62 50  40 -42 48  -32 -8 58  36 -2 56  14 10 30  14 10 58  36 -48 60  -38 -10 28  30 8 60 | 520  157  104  75  48  30  29  28  27  26  15 | 5.09  5.49  6.41  6.06  4.36  5.29  4.47  4.26  4.15  4.06  3.72 | Left  Left  Right  Right  Left  Right  Right  Right  Right  Left  Right | Medial Frontal Gyrus  Medial Frontal Gyrus  Superior Parietal Lobule  Inferior Parietal Lobule  Precentral Gyrus  Precentral Gyrus  Cingulate Gyrus  Medial Frontal Gyrus  Superior Parietal Lobule  Precentral Gyrus  Middle Frontal Gyrus |
|  |  |  |  |  |  |  |
| **Control** | 1  2  3  4  56  7  8  9 | -12 16 32  40 -42 44  16 -8 32  -50 22 24  4 10 52  -18 6 66  44 6 28  -32 22 0 | 1261  391  73  70  33  25  19  17 | 6.81  7.13  3.99  4.2  4.52  4.61  5.91  3.81 | Left  Right  Right  Left  Right  Left  Right  Left | Cingulate Gyrus  Inferior Parietal Lobule  Cingulate Gyrus  Inferior Frontal Gyrus  Medial Frontal Gyrus  Middle Frontal Gyrus  Precentral Gyrus  Claustrum |
| **4-Block**  **Early stopping:**  **Easy** | |  |  |  |  |  |
| **EPT** | 1  2  3  4  5  6 | 2 12 38  -8 -2 50  40 -46 46  -32 18 28  32 -68 40  -38 16 4 | 102  68  53  40  35  17 | 4.1  4.81  7.42  5.69  7.77  4.02 | Left  Left  Right  Left  Right  Left | Cingulate Gyrus  Cingulate Gyrus  Inferior Parietal Lobule  Middle Frontal Gyrus  Precuneus  Insula |
|  | |  |  |  |  |  |
| **Control** | 1  2  3  4  5  6  7  8  9 | 40 -46 46  -46 0 22  -12 16 34  -48 -38 40  -8 0 48  -48 30 28  20 -32 18  -20 -6 26  -34 16 8 | 566  466  259  233  127  121  68  27  19 | 9.51  5.78  6.36  4.36  5.27  4.06  4.08  3.5  3.95 | Right  Left  Left  Left  Left  Left  Right  Left  Left | Inferior Parietal Lobule  Inferior Frontal Gyrus  Cingulate Gyrus  Supramarginal Gyrus  Cingulate Gyrus  Middle Frontal Gyrus  Caudate  Caudate  Insula |
| **Hard** | |  |  |  |  |  |
| **EPT** | 1  2  3  4  5 | -6 -2 52  38 -46 48  -34 -8 60  30 -66 38  -22 12 28 | 546  132  69  57  57 | 6.17  7.97  5.41  5.14  4.6 | Left  Right  Left  Right  Left | Medial Frontal Gyrus  Inferior Parietal Lobule  Precentral Gyrus  Precuneus  Cingulate Gyrus |
|  | |  |  |  |  |  |
| **Control** | 1  2  3  4  5  6  7  8  9  10  11 | -40 10 24  -10 20 32  38 -46 48  -52 20 22  -32 -62 40  -48 -38 44  -14 -6 22  10 -12 28  -40 16 -8  40 10 26  8 8 24 | 424  313  312  89  84  82  50  31  23  19  15 | 7.06  6.39  6.47  4.9  4.13  5.12  3.89  4.11  4.59  4.75  3.99 | Left  Left  Right  Left  Left  Left  Left  Right  Left  Right  Right | Inferior Frontal Gyrus  Cingulate Gyrus  Inferior Parietal Lobule  Inferior Frontal Gyrus  Angular Gyrus  Inferior Parietal Lobule  Caudate  Cingulate Gyrus  Insula  Precentral Gyrus  Cingulate Gyrus |
| **Full duration:** | |  |  |  |  |  |
| **Easy** |  |  |  |  |  |  |
| **EPT** | 1  2  3  4 | -10 12 44  30 16 46  -34 12 26  -6 -2 64 | 167  36  15  15 | 5.59  4.47  6.52  5 | Left  Right  Left  Left | Cingulate Gyrus  Middle Frontal Gyrus  Precentral Gyrus  Medial Frontal Gyrus |
|  |  |  |  |  |  |  |
| **Control** | 1  2  3  4  5  6 | 40 -44 46  -38 2 26  -8 0 48  -48 -38 42  -48 30 30  22 -32 18 | 538  476  373  273  105  63 | 9.28  5.66  5.35  5.08  3.82  4.25 | Right  Left  Left  Left  Left  Right | IInferior Parietal Lobule  Precentral Gyrus  Cingulate Gyrus  Supramarginal Gyrus  Middle Frontal Gyrus  Caudate |
| **Hard** |  |  |  |  |  |  |
| **EPT** | 1  2  3 | -12 14 34  38 -46 48  4 14 56 | 293  63  57 | 8.41  8.36  6.43 | Left  Right  Right | Cingulate Gyrus  Inferior Parietal Lobule  Superior Frontal Gyrus |
|  |  |  |  |  |  |  |
| **Control** | 1  2  3  4  5  6  7  8  9  10  11  12 | -40 10 24  38 -46 48  -12 14 34  -16 -6 22  -32 -60 40  -54 20 22  -48 -38 44  16 -22 18  -40 16 -6  40 10 26  -6 10 50  20 -6 28 | 438  303  287  136  92  77  75  70  39  27  26  16 | 7.02  6.64  7.36  4.58  4.2  5.56  5.02  3.92  5.05  4.96  4.18  3.95 | Left  Right  Left  Left  Left  Left  Left  Right  Left  Right  Left  Right | Inferior Frontal Gyrus  Inferior Parietal Lobule  Cingulate Gyrus  Caudate  Angular Gyrus  Inferior Frontal Gyrus  Inferior Parietal Lobule  Thalamus  Insula  Precentral Gyrus  Medial Frontal Gyrus  Caudate |
